# FXR shapes an immunosuppressive microenvironment in PD-L1^lo/–^ non-small cell lung cancer by upregulating HVEM

**DOI:** 10.1172/jci.insight.190716

**Published:** 2025-09-23

**Authors:** Xiaolong Xu, Bin Shang, Hancheng Wu, Xiuye Jin, Junren Wang, Jing Li, Daowei Li, Bin Liang, Xingguang Wang, Lili Su, Wenjie You, Shujuan Jiang

**Affiliations:** 1 Department of Respiratory and Critical Care Medicine, Shandong Provincial Hospital affiliated to Shandong First Medical University, Jinan, Shandong, China.; 2 Department of Respiratory and Critical Care Medicine, Shandong Provincial Hospital, Shandong University, Jinan, Shandong, China.; 3 Department of Respiratory and Critical Care Medicine, the Second Hospital of Shandong University, Jinan, Shandong, China.; 4 Department of Thoracic Surgery, Shandong Provincial Hospital affiliated to Shandong First Medical University, Jinan, Shandong, China.; 5 Department of Thoracic Surgery, Shandong Provincial Hospital, Shandong University, Jinan, Shandong, China.; 6 Shandong Key Laboratory of Infectious Respiratory Disease, Jinan, Shandong, China.; 7 Medical Science and Technology Innovation Center, Shandong First Medical University & Shandong Academy of Medical Sciences, Jinan, Shandong, China.; 8 Department of Respiratory and Critical Care Medicine, Xi’an Chest Hospital, Shanxi, China.; 9 Department of Respiratory and Critical Care Medicine, Shandong Provincial Public Health Clinical Center, Jinan, Shandong, China.; 10 Departments of Thoracic Surgery and State Key Laboratory of Genetic Engineering, Fudan University Shanghai Cancer Center, Shanghai, China.

**Keywords:** Immunology, Oncology, Cancer immunotherapy, Lung cancer

## Abstract

Immune checkpoint therapy has changed cancer treatment, including non-small cell lung cancer (NSCLC). The unresponsiveness of PD-L1^lo/–^ tumors to anti–PD-1/PD-L1 immunotherapy is attributed to alternative immune evasion mechanisms that remain elusive. We previously reported that farnesoid X receptor (FXR) was increased in PD-L1^lo/–^ NSCLC. Herein, we found that immune checkpoint HVEM was positively correlated with FXR but inversely correlated with PD-L1 in NSCLC. HVEM was highly expressed in FXR^hi^PD-L1^lo^ NSCLC. Consistently, clinically relevant FXR antagonist dose-dependently inhibited HVEM expression in NSCLC. FXR inhibited cytokine production and cytotoxicity of cocultured CD8^+^ T cells in vitro, and it shaped an immunosuppressive tumor microenvironment (TME) in mouse tumors in vivo through the HVEM/BTLA pathway. Clinical investigations show that the FXR/HVEM axis was associated with immunoevasive TME and inferior survival outcomes in patients with NSCLC. Mechanistically, FXR upregulated HVEM via transcriptional activation, intracellular Akt, Erk1/2 and STAT3 signals, and G1/S cycle progression in NSCLC cells. In vivo treatment experiments demonstrated that anti-BTLA immunotherapy reinvigorated antitumor immunity in TME, resulting in enhanced tumor inhibition and survival improvement in FXR^hi^PD-L1^lo^ mouse Lewis lung carcinomas. In summary, our findings establish the FXR/HVEM axis as an immune evasion mechanism in PD-L1^lo/–^ NSCLC, providing translational implications for future immunotherapy in this subgroup of patients.

## Abstract 2

FXR-HVEM axis serves as an immune evasion mechanism in PD-L1^low/negative^ non-small cell lung cancer, which can be targeted by HVEM/BTLA blockade to evoke antitumor immunity.

## Graphical abstract

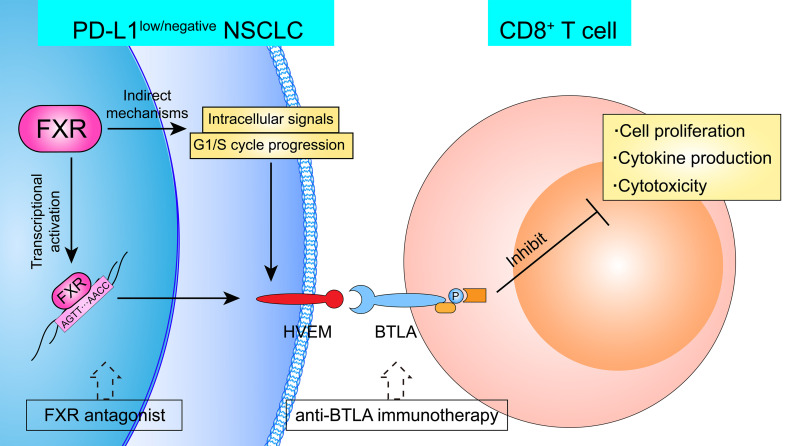

## Introduction

Non-small cell lung cancer (NSCLC), which makes up nearly 85% of patients with lung cancer, is one of the major causes of tumor-associated mortality globally (1, 2). Although therapeutic strategies have undergone dramatic advances in the past 20 years, the prognosis of NSCLC is still discouraging, with the 5-year overall survival (OS) rate below 20% (3). Recently, immune checkpoint therapy, which was designed to inhibit programmed death-1 (PD-1) or the ligand PD-L1 to prevent T cell exhaustion and induce prolonged antitumor immunity, has revolutionized the treatment for advanced-stage NSCLC (4–6). Recent clinical trials have documented the therapeutic benefits of anti–PD-1/PD-L1 monotherapy in patients with NSCLC who have PD-L1^hi^ expression (7, 8). However, the responsiveness to anti–PD-1/PD-L1 agents is relatively dismal in patients with PD-L1^lo/–^ NSCLC, which could be attributed to alternative immunosuppressive pathways in PD-L1^lo/–^ tumors that remain poorly understood.

Farnesoid X receptor (FXR), which is commonly known as a bile acid nuclear receptor, is highly expressed in the small intestine, liver, kidneys, lung tissues, and blood vessels (9, 10). As a transcription factor, FXR can be activated by ligands to regulate target gene expression involved in bile acid, cholesterol homeostasis, glucose, and lipid metabolism via targeting the promoter region as a monomeric component or a heterodimer with retinoid X receptor (11, 12). Recently, mounting studies have uncovered a tissue-specific oncogene function for FXR in different types of cancers of the esophagus, pancreas, and more (13, 14). In a previous study, we showed that FXR was increased in NSCLC tissues and promoted NSCLC cell proliferation (15). In another study, we reported a negative correlation between the expression of FXR and PD-L1 in NSCLC (16). Notably, in FXR^hi^PD-L1^lo^ mouse Lewis lung carcinoma (LLC) tumors, FXR diminished the infiltrating immune cells, especially the cytotoxic CD8^+^ T cells in tumor microenvironment (TME) (16). However, the detailed mechanism for FXR-suppressed antitumor CD8^+^ T cells in NSCLC has never been illustrated before.

Herpes virus entry mediator (HVEM), formerly referred to as tumor necrosis factor receptor superfamily 14 (*TNFRSF14*), represents another immune checkpoint protein that is expressed on lymphocytes, DCs, epithelial cells, mesenchymal cells, and more (17, 18). HVEM functions either as a costimulatory or as a coinhibitory signaling molecule depending on the binding partner. When HVEM interacts with LIGHT or another partner lymphotoxin-α, a stimulating signal is transmitted into T cells. Conversely, the interaction of HVEM with B and T lymphocyte attenuator (BTLA) or CD160 produces an inhibitory effect in proliferation or cytokine secretion of T lymphocytes (19). Despite the complexity, the overall feature of HVEM is immunosuppressive, since HVEM^–/–^ T cells exhibited a notable proliferation and cytokine production (20). This confers an increased susceptibility to MOG-triggered experimental autoimmune encephalopathy in HVEM^–/–^ mice. Aberrant expression of HVEM has recently been found to correlate with adverse prognosis and immune evasion in a variety of solid tumors (21–23). Regarding NSCLC, Ren et al. discovered that high expression of HVEM correlated significantly with metastasis of N2 lymph node in patients with NSCLC (24). Furthermore, they found a negative correlation between the expression of HVEM and PD-L1 in patients with NSCLC (24), indicating that HVEM may produce a suppressive effect to impair immunosurveillance in PD-L1^lo/–^ tumors. Given the inverse relationship in the distributions of FXR and PD-L1 in patients with NSCLC, there has been an increasing interest to explore whether FXR correlates with HVEM expression in NSCLC samples. HVEM may serve as an underlying mechanism — and, thus, a therapeutic immune target — in FXR-induced immunosuppressive TME of PD-L1^lo/–^ NSCLC.

Consequently, this work was aimed at finding out the correlation between FXR and HVEM in NSCLC tissues as well as the regulation of FXR on HVEM expression. In addition, the functional significance and molecular mechanisms of FXR-regulated HVEM were characterized. The clinical implications of the immunosuppressive FXR/HVEM axis were evaluated in a cohort of patients with NSCLC. The therapeutic potential of anti-BTLA immunotherapy, as well as the corresponding changes of TME, in the FXR^hi^PD-L1^lo^ NSCLC subtype were assessed using a syngeneic mouse tumor model.

## Results

### HVEM is positively correlated with FXR but is inversely correlated with PD-L1 in NSCLC.

**Figure 1:**
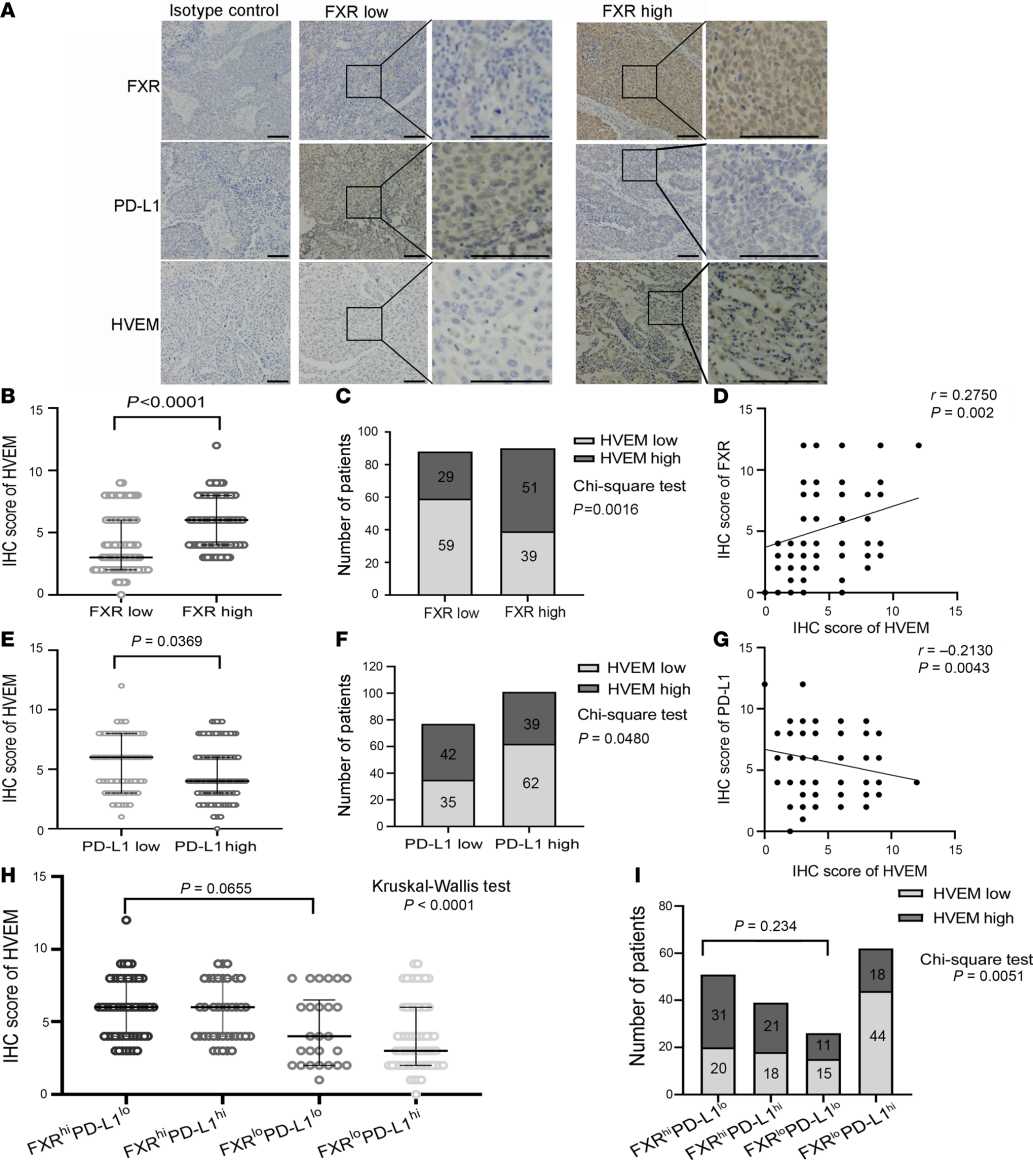
HVEM is positively correlated with FXR, but inversely correlated with PD-L1 in NSCLC. A total of 178 clinical NSCLC samples were subjected to IHC staining. (**A**) Representative images showing the expression of FXR, PD-L1, and HVEM in serial sections of FXR^hi^ vs. FXR^lo^ NSCLC samples at low (×80) and high (×320) magnifications. Nonspecific mouse or rabbit IgG was used as an isotype control antibody. Scale bar: 50 μm. (**B**) IHC score of HVEM in patients with NSCLC with FXR^hi^ vs. FXR^lo^ profiles (*P* < 0.0001, Mann-Whitney *U* test). (**C**) The number of HVEM high or low tumors in patients with NSCLC with FXR^hi^ vs. FXR^lo^ profiles (*P* = 0.0016, χ^2^ test). (**D**) Spearman’s rank correlation test showed a significant positive correlation between the expression of FXR and HVEM in 178 clinical NSCLC samples (*r* = 0.275, *P* = 0.002). (**E**) IHC score of HVEM in patients with NSCLC with PD-L1^hi^ vs. PD-L1^lo^ profiles (*P* = 0.0369, Mann-Whitney *U* test). (**F**) The number of HVEM high or low tumors in patients with NSCLC with PD-L1^hi^ vs. PD-L1^lo^ profiles (*P* = 0.048, χ^2^ test). (**G**) Spearman’s rank correlation test revealed a significant inverse correlation between the expression of PD-L1 and HVEM in 178 NSCLC samples (*r* = –0.213, *P* = 0.0043). (**H**) IHC score of HVEM in NSCLC samples divided into 4 subgroups based on FXR and PD-L1 expression (FXR^hi^PD-L1^lo^ vs. FXR^lo^PD-L1^lo^, *P* = 0.0655, Kruskal-Wallis rank sum test followed by Dunnett’s post hoc test). (**I**) The number of HVEM high or low tumors in NSCLC samples divided into 4 subgroups based on FXR and PD-L1 expression (FXR^hi^PD-L1^lo^ vs. FXR^lo^PD-L1^lo^, *P* = 0.234, χ^2^ test followed by Bonferroni post hoc test). In **B**, **E**, and **H**, the line and error bars represent the median and interquartile range.

First, to determine whether FXR correlates with HVEM expression in NSCLC, we examined clinical NSCLC samples and analyzed public datasets from The Cancer Genome Atlas (TCGA). Nuclear staining of FXR and membrane staining of HVEM were scored in respect to their subcellular locations in tumor cells (19, 25). As shown in Figure 1, A and B, HVEM was significantly higher in patients with FXR^hi^ NSCLC as compared with patients with FXR^lo^ NSCLC. χ^2^ analysis found that the mean percentage of HVEM^hi^ tumors was significantly increased in patients with FXR^hi^ NSCLC than in those with FXR^lo^ profiles (Figure 1C). Additionally, Spearman’s correlation test discovered a significant positive correlation between the expression of FXR and HVEM in clinical NSCLC cohort (Figure 1D). In order to validate our findings, we analyzed the available datasets of TCGA. There was a significant positive correlation between *NR1H4* (gene that encodes FXR) and *TNFRSF14* (gene that encodes HVEM) expression in 994 NSCLC samples of TCGA cohort (Supplemental Figure 1A; supplemental material available online with this article; https://doi.org/10.1172/jci.insight.190716DS1). Subgroup analysis revealed a similar positive correlation between the gene expression of *NR1H4* and *TNFRSF14* either in 510 adenocarcinoma or in 484 squamous cell carcinoma samples (Supplemental Figure 1, B and C). Collectively, these results indicate that FXR is positively correlated with the expression of HVEM in NSCLC.

To date, IHC-based detection of PD-L1 on tumor cells is the best predictive marker for anti–PD-1/PD-L1 immunotherapy in treating NSCLC (26). We then examined the correlation between PD-L1 and HVEM, and we showed that both the expression level of HVEM and the percentage of HVEM^hi^ tumors were significantly increased in patients with PD-L1^lo^ NSCLC as compared with PD-L1^hi^ patients (Figure 1, E and F). Importantly, Spearman’s correlation test discovered a significant inverse correlation between the expression of HVEM and PD-L1 in NSCLC samples (Figure 1G), suggesting that the immune checkpoints HVEM and PD-L1 are mutually exclusive. Furthermore, the clinical NSCLC samples were stratified according to FXR and PD-L1 status. When we paid attention to tumors with PD-L1^lo^ profiles, high FXR-expressing NSCLC (characterized by FXR^hi^PD-L1^lo^) had a nonsignificant higher level of HVEM, as well as a higher percentage of HVEM^hi^ tumors, than low FXR-expressing patients with NSCLC (characterized by FXR^lo^PD-L1^lo^) (Figure 1, H and I). This suggests to us that HVEM may be a key immuosuppressive factor in this subgroup (characterized by FXR^hi^PD-L1^lo^) of patients with PD-L1^lo/–^ NSCLC.

### FXR upregulates HVEM expression in NSCLC cells.

**Figure 2:**
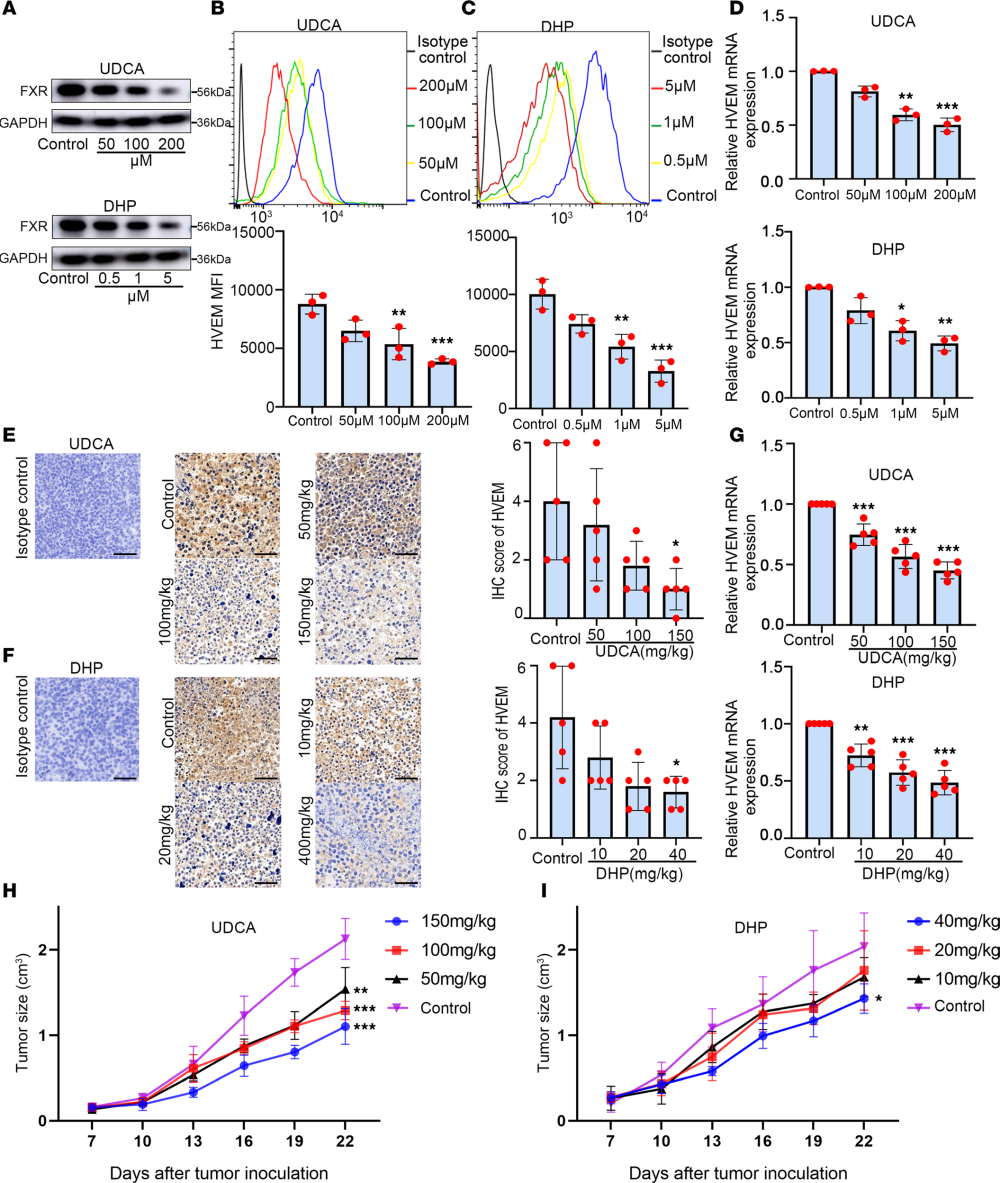
FXR antagonists dose-dependently inhibit HVEM expression in NSCLC. A549 cells were treated with concentration gradients of UDCA (0, 50, 100, and 200 μM) and DHP (0, 0.5, 1, and 5 μM) for 48 h. (**A**) The protein levels of FXR in A549 cells were examined by Western blotting. (**B** and **C**) Representative histograms and mean fluorescence intensity (MFI) quantifications for HVEM membrane staining in A549 cells treated with UDCA (**B**) and DHP (**C**) were analyzed by flow cytometry. (**D**) Relative mRNA levels of HVEM in A549 cells were examined by q-PCR. β-Actin served as an internal control. C57BL/6 mice were inoculated s.c. with 1 × 10^6^ LLC cells, and injected i.p. with increasing doses of UDCA (0, 50, 100, and 150 mg/kg) and DHP (0, 10, 20, and 40 mg/kg) every 3 days for 15 days when the tumor volume reached ~100 mm^3^. (**E** and **F**) Representative IHC images (magnification, ×200) and IHC score of HVEM expression in mouse LLC tumors treated with UDCA (**E**) and DHP (**F**) are shown. Nonspecific rabbit IgG was used as an isotype control antibody. Scale bar: 50 μm. (**G**) Relative mRNA levels of HVEM in mouse LLC tumors of each group were compared. (**H** and **I**) The tumor volume was monitored every 3 days after UDCA (**H**) and DHP (**I**) administration. Each experiment was conducted independently at least 3 times. Data are shown as mean ± SD from 3 biological replicates. For **E**–**I**, *n* = 5 mice/group. Statistical significance was assessed with 1-way ANOVA followed by Tukey’s post hoc test (**B**–**D** and **G**–**I**) or Kruskal-Wallis rank sum test followed by Dunnett’s post hoc test (**E** and **F**). *P < 0.05, **P < 0.01, ***P < 0.001, compared with control group.

Next, we explored whether the correlation between the levels of FXR and HVEM in patients with NSCLC implies a positive regulation. The functional relevance of FXR antagonists was determined in NSCLC cells. The results show that 3 antagonists of FXR, ursodeoxycholic acid (UDCA), 16-dehydropregnenolone (DHP), and Z-guggulsterone (Z-GS), could inhibit HVEM surface and mRNA expression in A549 cells in a dose-dependent manner (Figure 2, A–D, and Supplemental Figure 2, A–C). UDCA is a clinically used drug to treat human obesity, primary biliary cholangitis, and COVID-19 via decreasing FXR signaling (27–29). The effects of FXR antagonists on HVEM expression were also evaluated in vivo. Consistent with the above findings, UDCA, DHP, and Z-GS dose-dependently decreased HVEM membrane and mRNA expression in immune-competent mouse LLC tumor models (Figure 2, E–G, and Supplemental Figure 2, D–F). We found that both UDCA and DHP significantly reduced the in vivo tumor growth in mouse LLC tumor models in a dose-dependent manner (Figure 2, H and I). This is in line with our previous findings of Z-GS (30).

**Figure 3:**
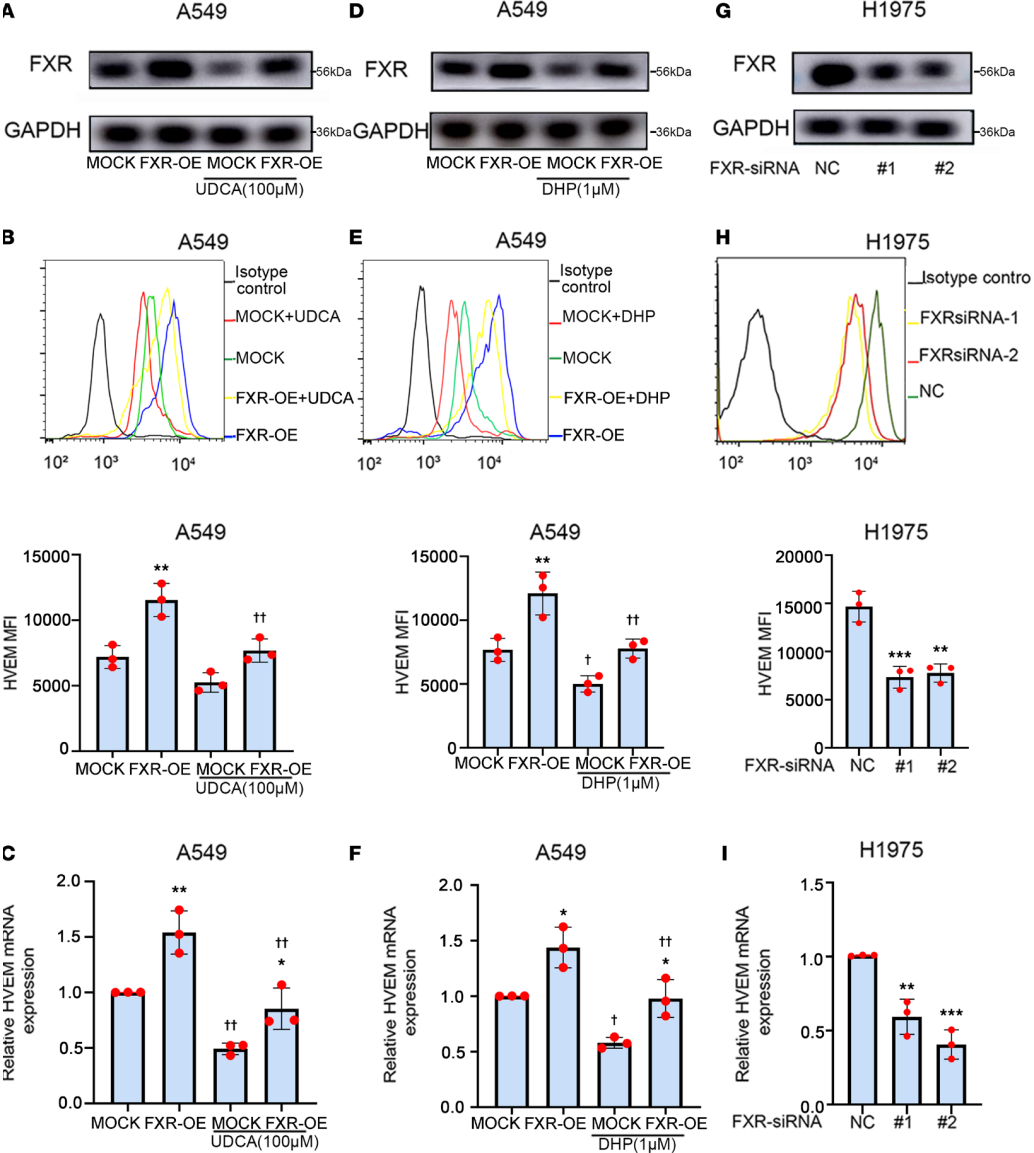
FXR upregulates HVEM expression in NSCLC cells. (**A**–**F**) A549 cells were infected with mock or FXR-overexpressed lentiviral vectors to establish stable cell lines, followed by treatment with 100 μM UDCA or 1 μM DHP for 48 h. (**G**–**I**) H1975 cells were transfected with NC- or FXR-siRNAs for 48 h. The protein levels of FXR in A549 stable cells treated with UDCA (**A**) or DHP (**D**) and H1975 cells (**G**) were examined by Western blotting. Representative histograms and MFI quantifications for HVEM membrane staining in A549 stable cells treated with UDCA (**B**) or DHP (**E**) and H1975 cells (**H**) were analyzed by flow cytometry. Relative mRNA levels of HVEM in A549 stable cells treated with UDCA (**C**) or DHP (**F**) and H1975 cells (**I**) were examined by qPCR. β-Actin served as an internal control. Each experiment was conducted independently at least 3 times. Data are shown as mean ± SD from 3 biological replicates. Statistical significance was assessed with 1-way ANOVA followed by Tukey’s post hoc test (**B**, **C**, **E**, **F**, **H**, and **I**). *P < 0.05, **P < 0.01, ***P < 0.001, compared with mock or NC group. In **B**, **C**, **E**, and **F**, ^†^*P* < 0.05, ^††^*P* < 0.01, compared with control group. OE, overexpression.

Moreover, the expression of HVEM in FXR-overexpressed or -silenced NSCLC cells was determined. The results indicate that stable overexpression of FXR led to an increased surface and mRNA expression of HVEM in A549 cells, which could be reversed by the treatment of either UDCA, DHP, or Z-GS (Figure 3, A–F, and Supplemental Figure 3, A–C). In comparison, FXR knockdown significantly downregulated both the cell surface protein and mRNA levels of HVEM in H1975 cells (Figure 3, G–I).

Collectively, these findings suggest that FXR upregulates HVEM expression in NSCLC cells.

### FXR inhibits cytokine production and cytotoxicity of CD8^+^ T cells through HVEM/BTLA checkpoint pathway.

**Figure 4:**
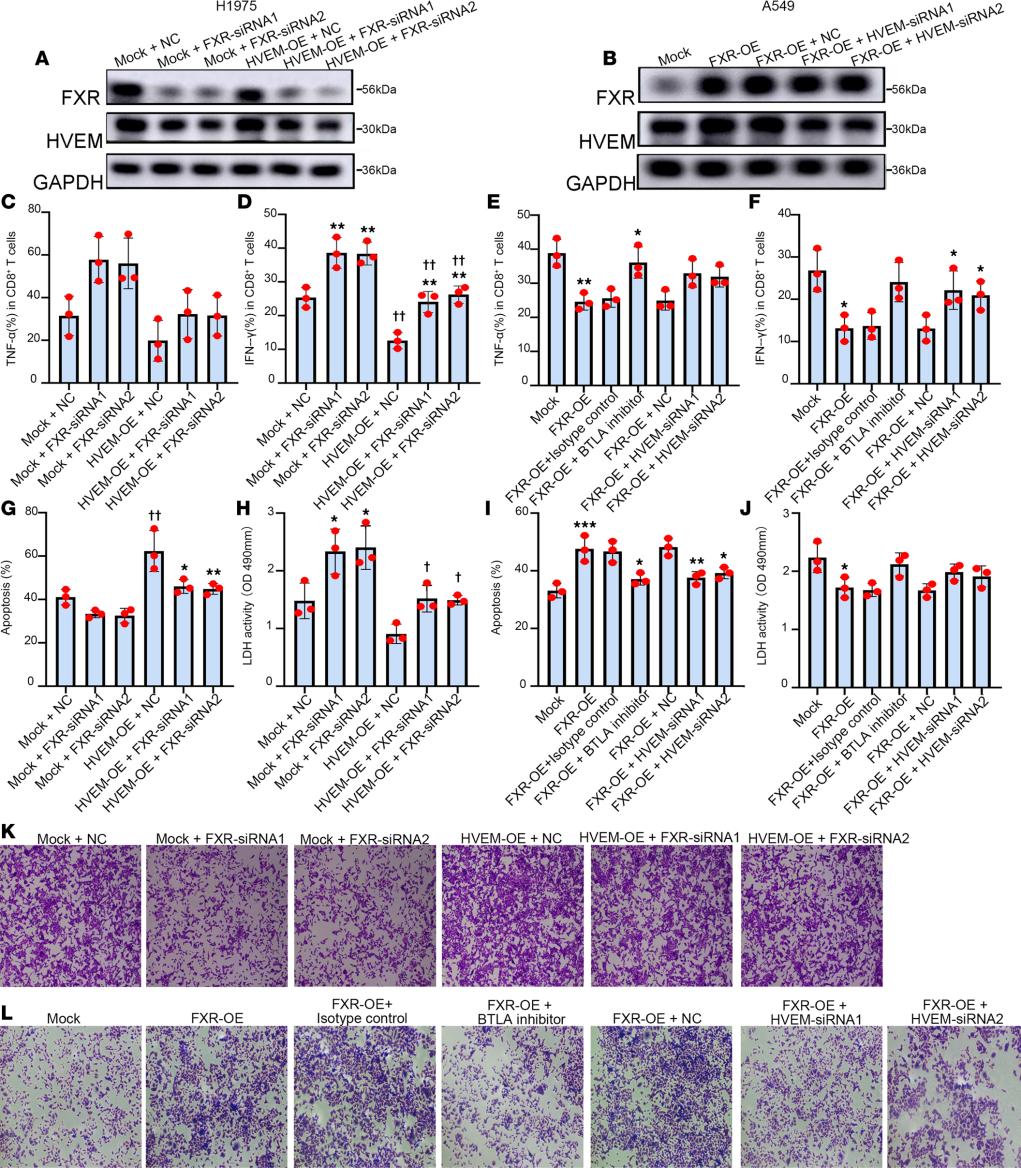
FXR inhibits cytokine production and cytotoxic activity of cocultured CD8^+^ T cells through HVEM/BTLA checkpoint pathway. Purified human CD8^+^ T cells were cocultured with HVEM-overexpressed H1975 stable cells which were transfected with FXR-siRNAs, or FXR-overexpressed A549 stable cells which were transfected with HVEM-siRNAs in the presence or absence of anti-BTLA antibody for 3 days. (**A** and **B**) The protein levels of FXR and HVEM in the cocultured H1975 stable cells (**A**) and A549 stable cells (**B**) were examined by Western blotting. (**C**–**F**) Intracellular TNF-α and IFN-γ expression levels in CD8^+^ T cells cocultured with H1975 stable cells (**C** and **D**) or A549 stable cells (**E** and **F**) was analyzed by flow cytometry. (**G** and **I**) The apoptotic rate of CD8^+^ T cells cocultured with H1975 stable cells (**G**) or A549 stable cells (**I**) was determined by flow cytometry using annexin V-FITC/PI staining assay. (**H** and **J**) The amounts of LDH released into the medium from the cocultured H1975 stable cells (**H**) or A549 stable cells (**J**) were measured. (**K** and **L**) The cell viability of cocultured H1975 stable cells (**K**) and A549 stable cells (**L**) was measured via crystal violet staining. Each experiment was conducted independently at least 3 times. Data are shown as mean ± SD from 3 biological replicates. Statistical significance was assessed with 1-way ANOVA followed by Tukey’s post hoc test (**C**–**J**). In **C**, **D**, **G**, and **H**, *P < 0.05, **P < 0.01, compared with NC group. ^†^*P* < 0.05, ^††^*P* < 0.01, compared with mock group. In **E**, **F**, **I**, and **J**, *P < 0.05, **P < 0.01, ***P < 0.001, compared with mock, isotype control, or NC group.

We then performed an in-depth study to evaluate the functional role of the FXR/HVEM axis in antitumor immunity in NSCLC. Since the cytotoxic CD8^+^ T cells are the central effectors in antitumor immunity (31), the effect of the FXR/HVEM axis on cytokine production and cytotoxicity of CD8^+^ T cells was evaluated in vitro. NSCLC cells with FXR/HVEM silencing or overexpression were cocultured with purified CD8^+^ T cells at a 1:25 target-to-effector cell ratio (Supplemental Figure 4, A and B), as described in our previous study (16). Cells were treated with or without anti-BTLA, a specific monoclonal antibody targeting HVEM/BTLA checkpoint pathway (32). The results show that knockdown of FXR in H1975 resulted in an increased proportion of IFN-γ^+^ or TNF-α^+^ cocultured CD8^+^ T cells, which was largely compromised by HVEM overexpression (Figure 4, A, C and D, and Supplemental Figure 4C). Conversely, enforced FXR expression in A549 significantly decreased the number of IFN-γ^+^ or TNF-α^+^ CD8^+^ T cells in cocultures (Figure 4, B, E, and F, and Supplemental Figure 4D). This decrease was reversed with the treatment of either anti-BTLA antibody or HVEM small interfering RNAs (siRNAs) (Figure 4, B, E, and F, and Supplemental Figure 4D). An opposite trend was observed for the apoptotic rate of cocultured CD8^+^ T cells (Figure 4, G and I, and Supplemental Figure 4, E and F). The cytotoxicity evoked by CD8^+^ T cells was also evaluated. We detected an enhanced cytotoxic activity of CD8^+^ T cells cocultured with FXR-silenced H1975 cells, as evidenced by the significantly increased supernatant lactate dehydrogenase (LDH) and the decreased cell viability in cocultured H1975 cells (Figure 4, H and K). Cytotoxic activity of CD8^+^ T cells was weakened by the introduction of HVEM in H1975 cells (Figure 4, H and K). In comparison, CD8^+^ T cells cocultured with FXR-overexpressed A549 led to a significantly decreased LDH release and increased cell viability in cocultured A549 cells (Figure 4, J and L). This effect was markedly abrogated by HVEM knockdown or the addition of anti-BTLA antibody in cocultures (Figure 4, J and L). These data collectively suggest that FXR inhibits cytokine production and cytotoxicity of cocultured CD8^+^ T cells through HVEM/BTLA checkpoint pathway.

### The FXR/HVEM axis is associated with immunoevasive TME and poor survival prognosis in patients with NSCLC.

**Figure 5:**
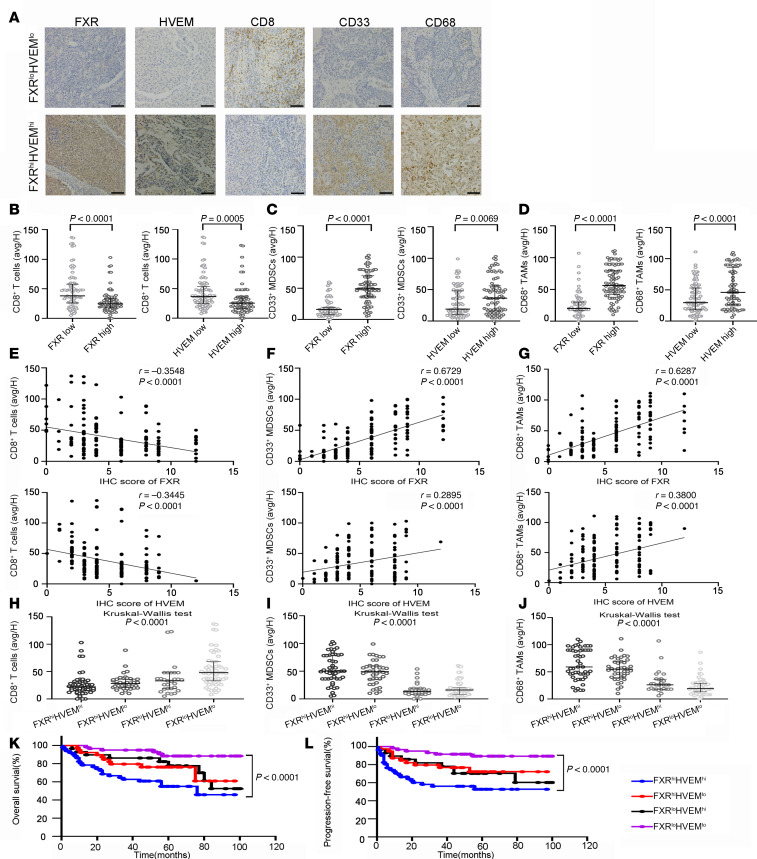
Correlation of FXR/HVEM axis with tumor immune infiltration and clinical prognosis in NSCLC. The enrolled 178 NSCLC samples in Figure 1 were subjected to IHC staining of CD8, CD33 and CD68. (**A**) Representative images showing FXR, HVEM, CD8, CD33, and CD68 expression in patients with NSCLC with FXR^hi^HVEM^hi^ vs. FXR^lo^HVEM^lo^ profiles (magnification, ×80). Nonspecific mouse or rabbit IgG was used as an isotype control antibody. Scale bar: 50 μm. (**B**–**D**) The number of infiltrating CD8^+^ T cells (**B**), CD33^+^ MDSCs (**C**), and CD68^+^ TAMs (**D**) in FXR^hi^ NSCLC vs. FXR^lo^ NSCLC (all *P* < 0.0001, Mann-Whitney *U* test; left graphs), or in HVEM^hi^ NSCLC vs. HVEM^lo^ NSCLC (*P* = 0.0005, 0.0069, < 0.0001, respectively, Mann-Whitney *U* test; right graphs). (**E**–**G**) Correlation analysis between the expression levels of FXR (upper graphs) and HVEM (lower graphs) and the proportions of infiltrating CD8^+^ T cells (**E**), CD33^+^ MDSCs (**F**) and CD68^+^ TAMs (**G**) in 178 NSCLC samples. Spearman’s rank correlation coefficients and statistical significance are shown. (**H**–**J**) The number of infiltrating CD8^+^ T cells (**H**), CD33^+^ MDSCs (**I**), and CD68^+^ TAMs (**J**) in NSCLC samples divided into 4 subgroups based on FXR and HVEM expression (all *P* < 0.0001, Kruskal-Wallis rank sum test). (**K** and **L**) Kaplan-Meier survival curves for OS (**K**, *P* < 0.0001, log-rank test) and PFS (**L**, *P* < 0.0001, log-rank test) in patients with NSCLC according to FXR and HVEM levels. In **B**–**D** and **H**–**J**, data are shown as mean ± SD.

To examine the clinical significance of the FXR/HVEM axis’s immunosuppressive effect, we evaluated tumor immune infiltration by IHC in the same cohort of patients with NSCLC. As expected, the number of infiltrating CD8^+^ T cells was significantly lower in FXR^hi^ NSCLC and HVEM^hi^ NSCLC, as compared with the FXR^lo^ subgroup and HVEM^lo^ subgroup, respectively (Figure 5, A and B, and Supplemental Figure 5). In contrast, a significant increase of immunosuppressive myeloid-derived suppressor cells (MDSCs) and tumor-associated macrophages (TAMs) were observed in FXR^hi^ NSCLC and HVEM^hi^ NSCLC as compared with those with FXR^lo^ profile or HVEM^lo^ profile, respectively (Figure 5, A, C, and D). The correlations between the levels of tumoral FXR and HVEM, and those immune infiltrations were analyzed. We found that both the expression levels of FXR and HVEM were inversely correlated with infiltrating CD8^+^ T cells in clinical NSCLC samples (Figure 5E). On the contrary, we observed a significant positive correlation either between the expression level of FXR and the proportions of MDSCs and TAMs, or between the expression of HVEM and the proportions of MDSCs and TAMs in the same cohort (Figure 5, F and G). Those clinical NSCLC samples were further stratified by FXR and HVEM expression levels. Consistently, immune composition data indicate that patients with high tumoral FXR and HVEM expression levels (FXR^hi^HVEM^hi^) had the lowest number of infiltrating CD8^+^ T cells and the highest number of infiltrating MDSCs and TAMs among all NSCLC subgroups (Figure 5, H–J). Investigation of the prognosis demonstrated that the FXR^hi^HVEM^hi^ subgroup was associated with the poorest progression-free survival (PFS) and OS among all NSCLC patient subgroups (Figure 5, K and L). These findings clearly suggest that the FXR/HVEM axis is correlated with immunoevasive TME and adverse clinical prognosis in patients with NSCLC.

### Molecular mechanisms accounting for FXR-upregulated HVEM in NSCLC.

**Figure 6:**
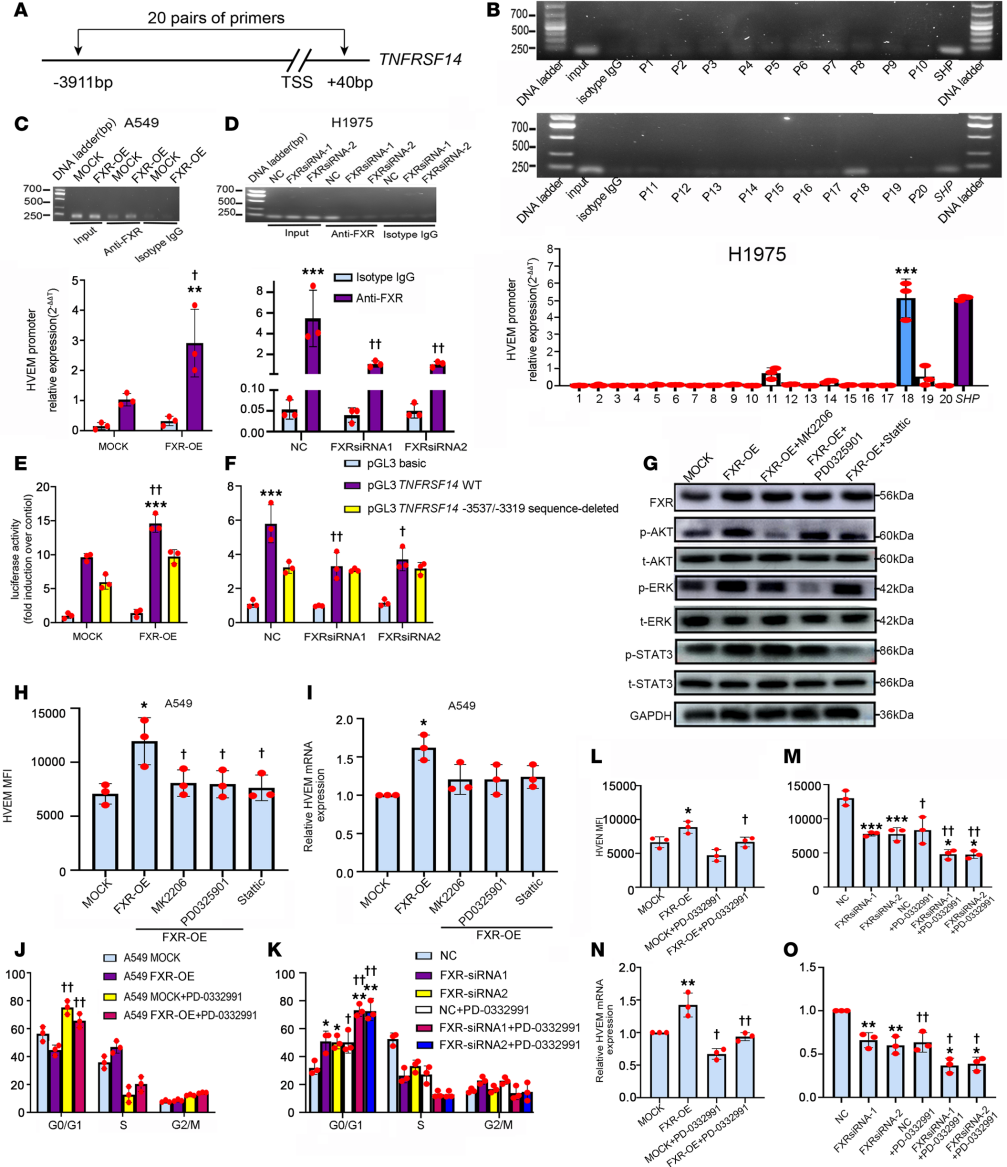
Molecular mechanisms by which FXR upregulates HVEM in NSCLC. (**A**) The region covered by 20 pairs of ChIP-qPCR primers in human TNFRSF14 promoter. (**B**–**D**) ChIP was performed in H1975 using the above ChIP-qPCR primers (**B**) or in FXR-overexpressed A549 (**C**) and FXR-silenced H1975 (**D**) using Primer 18 covering -3,537/-3,319 sequence. Representative gels (upper panels) and enrichments of FXR in each examined region (lower graphs) are shown. Input: non-immunoprecipitated chromatin. Negative control: isotype IgG. Positive control: primers covering FXR-binding site in SHP promoter. (**E** and **F**) FXR-overexpressed A549 (**E**) and FXR-silenced H1975 (**F**) were cotransfected with a WT or –3,537/–3,319 sequence-deleted TNFRSF14 promoter vector to examine luciferase activity. Negative control: basic vector. (**G**–**I**) FXR-overexpressed A549 was exposed to 0.3 µM MK2206, 0.3 µM PD0325901, or 4 µM Stattic for 48 h. (**G**) The expression of FXR, p-Akt (Ser473), t-Akt, p-Erk1/2 (Thr202/Tyr204), t-Erk1/2, p-STAT3 (Tyr705), and t-STAT3 were examined by Western blotting. (**H**) MFI quantifications of HVEM were analyzed by flow cytometry. (**I**) Relative HVEM mRNA levels were examined by qPCR. (**J**–**O**) FXR-overexpressed A549 and FXR-silenced H1975 were treated with 0.5 µM PD0332991 for 48 h. Cell cycle distributions and MFI quantifications of HVEM in A549 (**J** and **L**) and H1975 (**K** and **M**) were analyzed by flow cytometry. Relative HVEM mRNA levels in A549 (**N**) and H1975 (**O**) were examined by qPCR. Data are shown as mean ± SD from 3 biological replicates. Statistics were assessed with Student *t* test (**C** and **E**) or 1-way ANOVA followed by Tukey’s post hoc test (**B**, **D**, **F**, and **H**–**O**). In **B**, ***P < 0.001, compared with Primer 1–17, 19, and 20. In **C**–**F**, **P < 0.01, ***P < 0.001, compared with isotype IgG or –3,537/–3,319 sequence-deleted TNFRSF14 promoter vector. ^†^*P* < 0.05, ^††^*P* < 0.01, compared with mock or NC. In **H**–**O**, *P < 0.05, **P < 0.01, ***P < 0.001, compared with mock or NC. ^†^*P* < 0.05, ^††^ *P* < 0.01, compared with control group.

The mechanisms underlying FXR-induced HVEM in NSCLC were subsequently investigated. Considering that FXR is a transcription factor (11, 12), we examined the function of FXR in transcriptional control of *TNFRSF14* expression in NSCLC cells. Twenty pairs of primers were designed to cover the promoter region of *TNFRSF14* from –3,911 to +40 bp upstream of transcription start site (TSS) (Figure 6A and Supplemental Table 1). Primers corresponding to the FXR-binding site in the promoter of *SHP*, a downstream target gene of FXR, were used as positive control. ChIP analysis shows that the occupancy of FXR was significantly enriched in the sequence covered by Primer 18 from –3,537 to –3,319, as compared with the resting promoter region covered by other ChIP–quantitative PCR (ChIP-qPCR) primers (Figure 6, A and B). Notably, stable overexpression of FXR in A549 resulted in an increased binding of FXR to the nucleotide –3,537/–3,319 sequence in the promoter region of *TNFRSF14*, whereas the silencing of FXR significantly reduced FXR’s recruitment to this part of *TNFRSF14* promoter region in H1975 cells (Figure 6, C and D). Then, the functional relevance of FXR in *TNFRSF14* transcription was evaluated via luciferase reporter experiments by using constructs containing the *TNFRSF14* promoter with or without the nucleotide –3,537/–3,319 sequence. We found a significant increase of *TNFRSF14* transcription activity in FXR-overexpressed A549 and H1975 cells transfected with construct harboring the WT *TNFRSF14* promoter as compared with those with nucleotide –3,537/–3,319 sequence-deleted *TNFRSF14* promoter vector (Figure 6, E and F). Consistently, overexpression of FXR in A549 significantly promoted the transcription activity of the WT *TNFRSF14* promoter without extensively changing the transcription activity of the nucleotide –3,537/–3,319 sequence-deleted *TNFRSF14* promoter (Figure 6E). In comparison, FXR knockdown significantly decreased the transcription activity of the WT *TNFRSF14* promoter in H1975 cells, while the activity of the nucleotide –3,537/–3,319 sequence-deleted *TNFRSF14* promoter remained unchanged (Figure 6F). These data suggest that FXR transcriptionally activates HVEM expression in NSCLC cells.

We also examined the intracellular Akt, Erk1/2, and STAT3 signals, which were demonstrated to be downstream pathways of FXR (33). As shown in Figure 6G, the phosphorylated Akt (at Ser473), Erk1/2 (at Thr202/Tyr204), and STAT3 (at Tyr705) were increased in FXR-overexpressed A549 cells, implying the activation of intracellular Akt, Erk1/2, and STAT3. To determine whether these intracellular signals are involved in FXR-induced HVEM upregulation, we incubated NSCLC cells in the presence of selective Akt (MK2206), Erk1/2 (PD0325901), and STAT3 (Stattic) inhibitors. Since those signaling pathway inhibitors may affect cell proliferation and thereby yield an indirect effect on HVEM expression (34), the concentrations of MK2206, PD0325901, and Stattic that do not affect the proliferation of NSCLC cells were determined. As indicated, treatment with MK2206, PD0325901, or Stattic no more than 0.3 μM, 0.3 μM, and 4 μM, respectively, did not clearly inhibit the proliferation of A549 cells (Supplemental Figure 6A). Our results show that 0.3 μM MK2206, 0.3 μM PD0325901, and 4 μM Stattic effectively reduced the phosphorylation of Akt (Ser473), Erk1/2 (Thr202/Tyr204), and STAT3 (Tyr705) and partly abolished the increased HVEM surface and mRNA expression in FXR-overexpressed A549 cells (Figure 6, G–I). These data suggest that HVEM induction by FXR was partly explained by the activated Akt, Erk1/2, and STAT3 pathways in NSCLC.

Prior studies described the mutual regulation between immune checkpoints and cell proliferation in tumors (35, 36). We have reported that FXR promoted NSCLC cell proliferation via driving the cell cycle G1/S transition (15). Herein, we evaluated whether FXR-induced HVEM upregulation was due to cell cycle progression of NSCLC. As expected, overexpression of FXR decreased the proportion of A549 cells in G0/G1 phase (Figure 6J and Supplemental Figure 6B). This effect was reversed by the treatment of PD0332991, a specific CDK4/6 inhibitor (Figure 6J and Supplemental Figure 6B). Conversely, FXR silencing led to a significantly delayed G1/S transition in H1975 cells, which was further enhanced by PD0332991 (Figure 6K and Supplemental Figure 6C). The expression of HVEM was then determined. The results show that FXR-induced HVEM upregulation, either as protein at the cell surface or as mRNA, was significantly suppressed by PD0332991 in A549 cells (Figure 6, L and N). On the other hand, HVEM expression was further downregulated at both protein and mRNA levels in FXR-silenced H1975 cells treated with PD0332991 (Figure 6, M and O). These data suggest that FXR promotes cell cycle G1/S progression, which contributes to HVEM expression in NSCLC.

Taken together, these results demonstrate that FXR upregulates HVEM via transcriptional activation; intracellular Akt, Erk1/2, and STAT3 signaling pathways; and cell cycle G1/S progression in NSCLC.

### HVEM/BTLA blockade immunotherapy reactivates TME and exhibits antitumor activity against FXR^hi^PD-L1^lo^ mouse LLC tumors.

**Figure 7:**
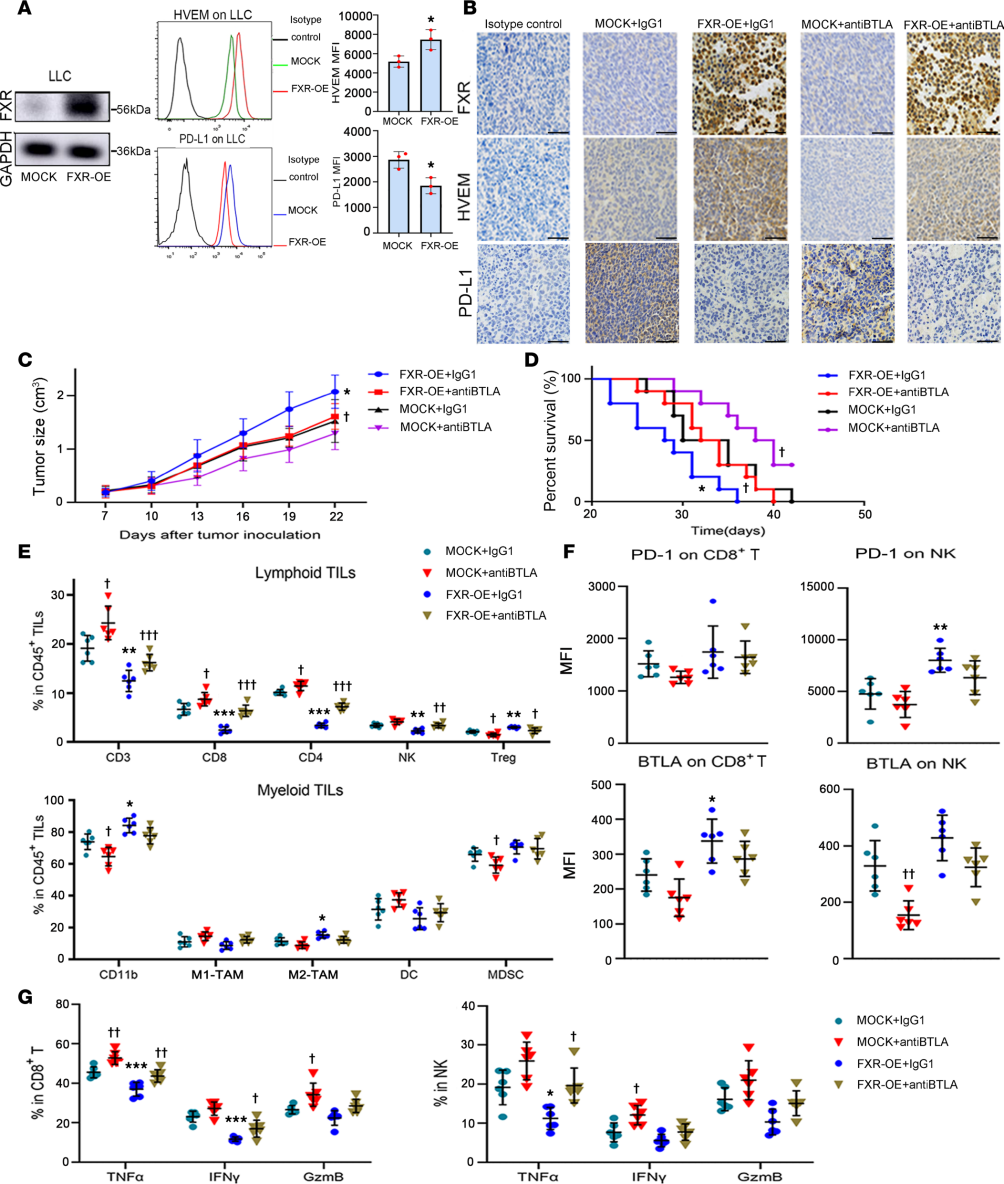
HVEM/BTLA blockade immunotherapy reactivates TME and produces antitumor activity against FXR^hi^PD-L1^lo^ mouse LLC tumors. (**A**) Mouse LLC cells were infected with mock or FXR-overexpressed lentiviral constructs to establish stable cells. The protein levels of FXR were examined using Western blotting (left panels). Representative histograms and MFI quantifications for HVEM and PD-L1 membrane staining were analyzed using flow cytometry (middle and right graphs). Each experiment was conducted independently at least 3 times. Data are shown as mean ± SD from 3 biological replicates. (**B**–**G**) C57BL/6 mice were inoculated s.c. with 1 × 10^6^ FXR-overexpressed or mock LLC cells, and injected i.p. with 200 μg anti–mouse BTLA/CD272 or mouse IgG1 isotype control every 3 days when the tumors reached a volume of ~100 mm^3^. (**B**) Representative IHC pictures showing the expression of FXR, HVEM, and PD-L1 in mouse LLC tumors of each group (magnification, ×200). Nonspecific rabbit IgG was used as an isotype control antibody. Scale bar: 50 μm. (**C**) Tumor volume was measured every 3 days after drug treatment. (**D**) Kaplan-Meier curves indicating percent survivals among each group. In **C** and **D**, *n* = 10 mice/group. (**E**) The percentages of tumor-infiltrating CD3^+^ T cells, CD8^+^ cytotoxic T cells, CD4^+^ Th cells, NK cells, Tregs, CD11b^+^ cells, MHC-II^+^ M1-TAMs, CD206^+^ M2-TAMs, DCs, and MDSCs in CD45^+^ cells in each group were determined using flow cytometry. (**F** and **G**) Surface staining of PD-1 and BTLA (**F**), as well as intracellular staining of TNF-α, IFN-γ, and GzmB (**G**), in tumor-infiltrating CD8^+^ cytotoxic T cells and NK cells of each group were examined using flow cytometry. In **E**–**G**, *n* = 6 mice/group. Data are presented as mean ± SD. Statistical significance was assessed with Student *t* test (**A**), log-rank test (**D**), or 1-way ANOVA followed by Tukey’s post hoc test (**C** and **E**–**G**). *P < 0.05, **P < 0.01, ***P < 0.001, compared with mock group. ^†^*P* < 0.05, ^††^*P* < 0.01, ^†††^*P* < 0.001, compared with isotype control. TIL, tumor-infiltrating lymphocyte.

In regard to the suppressive effect of FXR/HVEM axis on antitumor CD8^+^ T cells, we investigated whether targeting the immune checkpoint HVEM/BTLA complex could evoke antitumor immunity and produce antitumor activity in the context of FXR^hi^PD-L1^lo^ NSCLC in vivo. FXR-overexpressed or mock murine LLC cells were inoculated s.c. into C57BL/6 mice, which were then treated i.p. with anti-BTLA or isotype control antibodies. Consistent with our previous work (16), the downregulation of PD-L1 was recapitulated in FXR-overexpressed LLC cells both in vitro and in vivo, suggesting an FXR^hi^PD-L1^lo^ signature (Figure 7, A and B). As shown in Figure 7A, enforced FXR overexpression resulted in an increased HVEM surface expression in LLC cells. IHC staining confirmed the in vivo upregulation of HVEM in mouse LLC-FXR tumors, as compared with mock LLC tumors (Figure 7B). We observed a significantly increased tumor volume and a significantly worse OS in mice bearing LLC-FXR tumors as compared with those bearing LLC-Mock tumors (Figure 7, C and D). Importantly, anti-BTLA significantly decreased tumor growth and improved OS in LLC-FXR tumor–bearing mice, while this treatment was less effective against LLC-Mock tumors (Figure 7, C and D). These data suggest that the FXR^hi^PD-L1^lo^ phenotype confers a high sensitivity to HVEM/BTLA blockade immunotherapy in mouse LLC tumors.

To clarify the mechanism underlying the antitumor activity of BTLA inhibition, we examined immune cells in the TME of mouse LLC tumors via flow cytometry (Supplemental Figure 7A). The data show that mouse LLC-FXR tumors displayed significantly lower numbers of lymphoid cells, including cytotoxic CD8^+^ T cells, CD4^+^ Th cells, and NK cells in comparison with mock LLC tumors (Figure 7E and Supplemental Figure 7B). In contrast, the Tregs were significantly increased in LLC-FXR tumors as compared with mock LLC tumors. Analysis of myeloid cells revealed significantly increased infiltration of CD11b^+^ cells and CD206^+^ M2 phenotype TAMs (M2-TAMs), and a trend of decreased infiltration of MHC-II^+^ M1 phenotype TAMs (M1-TAMs) in LLC-FXR tumors compared with the mock group. This indicated an M1-to-M2 TAM polarization. As expected, we detected significantly increased infiltrations of CD3^+^ T cells, CD4^+^ Th cells, cytotoxic CD8^+^ T cells, and NK cells in mouse LLC-FXR tumors after receiving anti-BTLA antibody (Figure 7E and Supplemental Figure 7B). Conversely, a significant decline in the frequency of Tregs was detected in anti-BTLA–treated mouse LLC-FXR tumors. BTLA blockade also increased the infiltrating CD3^+^ T cells, CD4^+^ T cells, and CD8^+^ T cells, and it decreased the number of Tregs in mouse LLC-Mock tumors but to a lower degree in comparison with LLC-FXR tumors. As for the myeloid cells, anti-BTLA treatment resulted in a trend of decreased infiltration of CD11b^+^ cells and M2-TAMs and increased infiltration of M1-TAMs in mouse LLC-FXR tumors. This implies a repolarization of TAMs from an M2 to M1 phenotype. In comparison, only decreased CD11b^+^ cells and MDSCs were observed in mouse LLC-Mock tumors following the treatment with anti-BTLA.

The molecule features of infiltrating immune cells from mouse LLC tumors were characterized. PD-1 and BTLA represent 2 key immune inhibitory checkpoints in tumor immune evasion (37). The results show that mouse LLC-FXR tumors had significantly higher frequencies of PD-1^+^ NK cells and BTLA^+^CD8^+^ cytotoxic T cells than LLC-Mock tumors (Figure 7F and Supplemental Figure 7C). We observed a trend toward increased, but not significant, expression of PD-1 on CD8^+^ T cells and expression of BTLA on NK cells in LLC-FXR tumors as compared with mock tumors. This indicates an exhausted status of these 2 tumoricidal effector cells. In addition, CD8^+^ T cells and NK cells from LLC-FXR tumors exhibited a depressed effector function, as evidenced by significantly reduced TNF-α and IFN-γ in CD8^+^ T cells, and significantly reduced TNF-α in NK cells as compared with those from LLC-Mock tumors (Figure 7G and Supplemental Figure 7, D and E). Notably, BTLA blockade led to a nonsignificant downregulation of PD-1 and BTLA in NK cells from mouse LLC-FXR tumors (Figure 7F and Supplemental Figure 7C). CD8^+^ T cells in anti-BTLA–treated LLC-FXR tumors had a weak downregulation of PD-1 and BTLA. Moreover, we detected a significant concordant upregulation of TNF-α and IFN-γ in CD8^+^ T cells as well as an upregulation of TNF-α in NK cells in mouse LLC-FXR tumors after receiving anti-BTLA antibody (Figure 7G and Supplemental Figure 7, D and E). There was also a trend toward decreased PD-1 and BTLA either in CD8^+^ T cells or in NK cells, as well as increased TNF-α, IFN-γ, and granzyme B (GzmB) in CD8^+^ T cells and NK cells. This effect was seen to a lesser degree in anti-BTLA–treated mouse LLC-Mock tumors (Figure 7, F and G, and Supplemental Figure 7, C–E). In combination, the above data suggest that FXR shapes an immunosuppressive TME in FXR^hi^PD-L1^lo^ mouse LLC tumors, which is reactivated by the treatment of HVEM/BTLA blockade immunotherapy.

## Discussion

Tumor-infiltrating immune cells have been recognized as one of requisites for a response to immune checkpoint immunotherapy (38). Strategies to overcome the immunosuppressive TME should boost antitumor immunity and achieve tumor control (39). We showed in a previous study that FXR constructed an immunosuppressive TME in FXR^hi^PD-L1^lo^ NSCLC, shown as a decrease in cytotoxic CD8^+^ T cells (16). However, the detailed mechanism for FXR-suppressed antitumor CD8^+^ T cells is not yet understood. In this study, by analyzing the clinical NSCLC cohort and TCGA dataset, we discovered a positive correlation between the expression of FXR and HVEM in patients with NSCLC. Furthermore, HVEM was inversely correlated with PD-L1 in patients with NSCLC, and highly expressed in FXR^hi^PD-L1^lo^ NSCLC tumors. Accordingly, a clinically relevant FXR antagonist dose-dependently inhibited HVEM expression in NSCLC both in vitro and in vivo. Our results demonstrate that FXR upregulated HVEM expression via transcriptional activation, intracellular Akt, Erk1/2 and STAT3 signals, and cell cycle G1/S progression in NSCLC, thereby inhibiting the cytokine production and cytotoxic activity of tumor-infiltrating CD8^+^ T cells. Clinical study validated that the FXR/HVEM axis was correlated with an immunoevasive contexture and poor survival prognosis in patients with NSCLC. Despite the fact that available agents targeting HVEM/BTLA have shown encouraging results in epithelial ovarian carcinoma and chronic lymphocytic leukemia (40, 41), there is no published study to evaluate the therapeutic efficacy of HVEM/BTLA inhibition in NSCLC. This study originally demonstrated that anti-BTLA immunotherapy reactivated antitumor immune responses in TME, shown as the increased infiltration, activation, and effector functions of tumoricidal CD8^+^ T cells and NK cells. This led to an enhanced tumor control in FXR^hi^PD-L1^lo^ NSCLC. In this respect, this study provides a promising immunotherapy option for this subgroup (characterized by FXR^hi^PD-L1^lo^) of patients with PD-L1^lo/–^ NSCLC.

Recently, FXR has emerged as a tissue-specific oncogene that modulates a range of cancer-associated genes, such as COX-2, cyclin D1 and VEGF (13–15). Herein, our data suggest that FXR upregulated the tumorigenic HVEM both in vitro and in vivo, supporting the oncogenic functions of FXR in NSCLC. On the other hand, FXR has been documented in regulation of immunity in various diseases (10, 42). We showed in a previous work that FXR downregulated PD-L1 in NSCLC cells (16). Meanwhile, accumulating evidence found that FXR activation limited immune cell recruitment, decreased immunostimulatory cytokine and chemokine production, and restrained autoimmunity, while FXR deficiency or ablation produced an opposite effect, suggesting a general suppressive role for FXR in distinct immunological contexts (43–46). Consistently, this study shows that FXR overexpression created an immunosuppressive TME, characterized by the decreased infiltration of cytotoxic CD8^+^ T cells, CD4^+^ Th cells, and NK cells; increased frequencies of PD-1^+^ NK cells and BTLA^+^ CD8^+^ T cells; and reduced TNF-α, IFN-γ, and GzmB expression both in NK cells and CD8^+^ T cells, through HVEM/BTLA checkpoint pathway in FXR^hi^PD-L1^lo^ mouse LLC tumors. The immunosuppressive effects of the FXR/HVEM axis on tumor immune infiltrations were validated in clinical NSCLC samples. However, this study does not exclude the possibility that additional immune molecules or cells may favor FXR-dependent immune evasion in this setting, considering the increased Treg infiltration and M2-skewed TAM polarization in TME of mouse FXR^hi^PD-L1^lo^ LLC tumors. He et al. reported that FXR signaling was crucial for the immunosuppressive and antibacterial functions of MDSCs in neonatal sepsis (47). Therefore, FXR should be a more complicated regulator in TME, which is worthy of future investigation in other malignancies.

FXR functions as a transcription factor, which can be recruited to the promoter of downstream target genes to activate or repress their transcription (11, 12). Herein, we surveyed the promoter region of *TNFRSF14* by using 20 pairs of primers that amplify sequence from –3,911 to +40 upstream of TSS. Moreover, we showed that FXR increased the transcription of *TNFRSF14* through directly binding to the nucleotide –3,537/–3,319 sequence in the *TNFRSF14* promoter by ChIP and luciferase reporter assays. Notwithstanding the transcriptional regulation, FXR can also modulate a variety of cell signaling pathways, such as PI3K/Akt, MEK/Erk1/2, Jak/STAT, and Wnt/β-catenin in tumor cells (33). In this regard, we found that FXR overexpression in NSCLC led to the activation of intracellular Akt, Erk1/2, and STAT3 signals. What is noteworthy is that either MK2206, PD0325901, or Stattic treatment could partly block FXR-induced HVEM upregulation, suggesting another mechanism that FXR-induced HVEM is partly mediated by the intracellular Akt, Erk1/2, and STAT3 signals in NSCLC. FXR promoted NSCLC proliferation via driving the cell cycle G1/S transition (15). We then focused on the fitness of tumor cells, which is considered to be linked with immune checkpoint expression (35, 36). The data demonstrate that FXR induced the cell cycle G1/S progression, which contributed to the upregulation of HVEM in NSCLC cells. Although aberrant expression of HVEM has been reported to predict adverse clinical outcomes across many different tumor types (21–23), how HVEM expression in tumor cells is regulated remains elusive. This study provides molecular mechanisms that govern the expression of HVEM in NSCLC cells.

Immunotherapy designed to block the PD-1/PD-L1 axis has shown superior antitumor efficiency in patients with NSCLC with high PD-L1 expression (7, 8). While much of the recent efforts are geared toward combined strategies to improve clinical benefits, immunotherapeutic approaches are needed for those PD-L1^lo/–^ patients. Dissecting alternative immune evasion mechanisms in the context of PD-L1^lo/–^ tumors could be helpful to identify potential therapeutic targets. Indeed, early studies reported the immunsuppressive effects of several possible immune checkpoints, including CD276, Siglec-15, B7x, HHLA2, and PD-L2 in patients with PD-L1^lo/–^ cancer (48–51). However, the detailed mechanisms underlying immune suppression in PD-L1^lo/–^ tumors remain unclear. The current study showed a mutually exclusive expression pattern of HVEM and PD-L1 in NSCLC samples, which is in agreement with a front report (24). We observed that patients with FXR^hi^PD-L1^lo^ NSCLC exhibited a higher level of HVEM than patients with FXR^lo^PD-L1^lo^ NSCLC. Consistently, FXR antagonists, including the clinically used UDCA, dose-dependently inhibited HVEM expression in NSCLC both in vitro and in vivo. Our data further demonstrate that it was FXR-induced HVEM in NSCLC that exerted an immunosuppressive function on cocultured CD8^+^ T cells in vitro and contributed to the immunosuppressive TME in mouse LLC tumors in vivo. Using clinical NSCLC samples, we clarified the inverse correlations between tumoral FXR and HVEM and infiltrating CD8^+^ T cells as well as the positive correlations between tumoral FXR and HVEM and the proportions of MDSCs and TAMs. The FXR^hi^HVEM^hi^ subgroup had the lowest number of infiltrating CD8^+^ T cells, the highest number of infiltrating MDSCs and TAMs, and the poorest PFS and OS in NSCLC. Recent studies have initially explored the therapeutic potential of immunomodulatory agents targeting PD-1/PD-L1 alternative pathways, such as Siglec-15, CTLA-4, and even the PD-1/PD-L2 axis, for PD-L1^lo/–^ tumors (49, 52, 53). However, the results remain disappointing due to the poor understanding of their functions in cancer immunity and, more importantly, the lack of reliable predictors to identify the most likely responders to their blockade. Our in vivo treatment experiments show that targeting HVEM/BTLA pathway with anti-BTLA significantly decreased tumor growth and prolonged OS in FXR^hi^PD-L1^lo^ LLC tumor–bearing mice, in comparison with those bearing mock LLC tumors. More striking is the reactivation of TME in FXR^hi^PD-L1^lo^ mouse LLC tumors after anti-BTLA treatment, as shown by increased numbers of infiltrating cytotoxic CD8^+^ T cells, CD4^+^ T cells, and NK cells; decreased frequencies of Tregs; and restored activation and effector responses of infiltrating CD8^+^ T cells and NK cells. Currently, a humanized anti-BTLA (JS004) designed to target HVEM/BTLA is undergoing a clinical trial of phase I for advanced solid tumors, including gastric adenocarcinoma, esophageal squamous cell carcinoma, liver, cervical, and MSI-H colorectal cancers. This study warrants future clinical research of anti-BTLA immunotherapy to treat patients with PD-L1^lo/–^ NSCLC. While our results show that both UDCA and DHP dose-dependently reduced tumor growth in mouse LLC tumor models, we hypothesized that HVEM/BTLA blockade would be combined with FXR antagonists to provide additional therapeutic benefit in NSCLC, especially for the FXR^hi^PD-L1^lo^ subgroup.

There are several limitations to this study. Only s.c. tumor-bearing models with LLC cells were employed. Orthotopic transplantation models of diverse murine NSCLC tumors should be adopted. Moreover, since sex may be a biological variable that affects immunological responses to tumor antigens, establishment of in vivo studies to include more male and female mice will strengthen the conclusions of this study in the future.

In summary, the present study shows that HVEM was positively correlated with FXR and preferentially expressed in FXR^hi^PD-L1^lo^ NSCLC. FXR shaped an immunosuppressive TME in NSCLC by inducing HVEM expression through transcriptional activation, intracellular Akt, Erk1/2 and STAT3 signaling pathways, and G1/S cycle progression. Importantly, HVEM/BTLA blockade immunotherapy reinvigorated antitumor immunity in TME, thereby leading to an enhanced tumor control and improved survival in FXR^hi^PD-L1^lo^ mouse LLC tumors. This study offers insights into the mechanism of FXR-dependent immune evasion in NSCLC TME and supports the therapeutic potential of targeting the FXR/HVEM axis in this subset of patients with PD-L1^lo/–^ NSCLC.

## Methods

### Sex as a biological variable.

This study included male and female individual clinical samples. Similar findings were found for both sexes. Sex was not considered as a biological variable. For in vivo studies, only female mice were used based on practical considerations, such as immunity, aggressiveness, and welfare.

### Cell lines.

Human NSCLC A549 and H1975 cells as well as mouse LLC cells were all purchased from the American Type Culture Collection (ATCC) and grown in DMEM or RPMI-1640 medium with 10% FBS and 1% penicillin-streptomycin. The above cells were confirmed to be free of *Mycoplasma* and authenticated by the short tandem repeat (STR) method. All cells were passaged no more than 25 times after resuscitation. MK-2206 (HY-108232; MedChemExpress, NJ), PD0325901 (HY-10254; MedChemExpress), UDCA (HY-13771; MedChemExpress), DHP (sc-287310; Santa Cruz, CA), Z-GS (sc-204414; Santa Cruz), Stattic (abs812053; Absin, Shanghai, China), and Palbociclib (PD-0332991; S1116; Selleck, Shanghai, China) were free of endotoxins and were dissolved by using dimethyl sulfoxide (DMSO; 67-68-5; Sigma-Aldrich) according to manufacturer’s instructions.

### Patient samples.

From February 2015 to December 2020, primary NSCLC tumors were consecutively obtained from Asian Chinese patients who underwent surgery at the Department of Thoracic Surgery in Shandong Provincial Hospital (Jinan, China). Here were the inclusion criteria we followed: (a) complete resection with curative intent and (b) pathologically confirmed NSCLC by 2 independent pathologists. The exclusion criteria were: (a) neo-adjuvant chemotherapy or radiotherapy treatment and (b) medical history of other malignant tumors. The survival data were collected through medical records or telephone interviews. Finally, a total of 178 NSCLC samples were enrolled and made as formalin-fixed paraffin-embedded (FFPE) specimens. Peripheral whole blood samples were collected from patients newly diagnosed with NSCLC between October 2020 and April 2021 in Shandong Provincial Hospital.

### IHC analysis.

The FFPE NSCLC samples and mouse LLC tumors were stained with IHC as described previously (30). Primary antibodies to examine human FXR, HVEM, PD-L1, CD8, CD33, and CD68, and mouse FXR, HVEM and PD-L1 were as follows: anti-bile acid receptor *NR1H4* (1:100; ab187735; Abcam, Cambridge, UK), anti-HVEM/*TNFRSF14* (1:100; sc-365971; Santa Cruz), anti–PD-L1 (1:400; ab205921; Abcam), anti-CD8α (1:100; ab101500; Abcam), anti-CD33 (1:1000; ab270942; Abcam), anti-CD68 (1:3000; ab955; Abcam), anti-FXR1 (1:100; ab155124; Abcam), anti-HVEM/*TNFRSF14* (1:200; 10138-1-AP; Proteintech, IL) and anti–PD-L1 antibodies (1:100; 64988S; Cell Signaling Technology, Beverly, MA). For isotype controls, nonspecific rabbit and mouse IgG (ab172730 and ab37355; Abcam) were used at concentrations matched with the primary antibodies.

The results of IHC staining were scored independently and blindly by 2 skilled pathologists, and a final consensus was reached. Negative (score, 0), weak (score, 1), moderate (score, 2) and intense (score, 3) represent tumor cell staining intensity, respectively. 0% (score, 0), 1% to 25% (score, 1), 26% to 50% (score, 2), 51% to 75% (score, 3), and 76% to 100% (score, 4) represent the percentage of positive cells. The final IHC score was the multiplication result of intensity score and percentage score. In human NSCLC tissues, the levels of FXR, PD-L1, and HVEM were all categorized as low expression (score, 0–4) or high expression (score, 6–12), respectively, in accordance with our previous study (54). The infiltrations of CD8^+^ T cells, MDSCs and TAMs in NSCLC samples were evaluated as previously described (54).

### Cell counting kit-8 (CCK-8) assay.

Cell proliferation was measured using the CCK-8 assay. In brief, cells were incubated with CCK-8 solution (10 μL/well; CK04; Dojindo Molecular Technologies) in the dark at 37°C for 2 hours. The absorbance (OD) was measured at 490 nm using a Thermo Fisher Multiskan FC microplate reader.

### Cell cycle analysis.

Cells were harvested and fixed with 70% ice-cold ethanol. Then, the fixed cells were resuspended in propidium iodide/RNase staining buffer (550825; BD Pharmingen) for 30 minutes at room temperature in the dark. Flow cytometry was performed to analyze the cell cycle distributions.

### Western blotting.

Western blotting was carried out according to our preliminary publication (15). Primary antibodies were used at dilutions in compliance with the manufacturer’s recommendations: anti-HVEM/*TNFRSF14* antibody (sc-365971; Santa Cruz), anti-bile acid receptor *NR1H4* antibody (ab187735; Abcam), anti-*NR1H4* antibody (bs-12867R; Bioss, Beijing, China), anti-phosphorylated (p)-STAT3 (Tyr705) (9145S; Cell Signaling Technology), anti-STAT3 (12640S; Cell Signaling Technology), anti-p-Erk1/2 (Thr202/Tyr204) (4370S; Cell Signaling Technology), anti-Erk1/2 (4695S; Cell Signaling Technology), anti-p-Akt (Ser473) (9271S; Cell Signaling Technology), anti-Akt (4691S; Cell Signaling Technology) and anti-GAPDH antibodies (92310SF; Cell Signaling Technology).

### qPCR analysis.

qPCR was conducted in accordance with our previous study (30). The following primer sequences were used: 5′-AGTCCAGGTTATCGTGTGAAG-3′ (sense) and 5′-AGACACTTGCTTAGGCCATT-3′ (antisense) for human HVEM, 5′-TTGCTGATCCACATCTGCT-3′ (sense) and 5′-GACAGGATGCAGAAGGAGAT-3′ (antisense) for human β-actin, 5′-GTTCCTTGTGGGAGACGAGTG-3′ (sense) and 5′-CATGGGCGGTATATGTCTGTGG-3′ (antisense) for mouse HVEM, 5′-GGCATTGTTACCAACTGGGACGAC-3′ (sense) and 5′-CCAGAGGCATACAGGGACAGCACAG-3′ (antisense) for mouse β-actin.

### Lentiviral constructs and cell infection.

Lentiviral vectors were built by using the pCMV-*NR1H4*-PGK-PuroR vector, which harbors human *NR1H4* cDNA sequence (GenBank accession no. NM_005123.3); pcSLenti-EF1-PuroR-CMV-*TNFRSF14*-WPRE vector, which harbors human *TNFRSF14* cDNA sequence (GenBank accession no. NM_003820.4); or the pCMV-m*NR1H4*-PGK-PuroR vector, which contains mouse *NR1H4* cDNA sequence (GenBank accession no. NM_009108.2), from OBiO Biotechnology. Cell infection was performed by using lentiviral plasmids according to our preliminary publication (15).

### siRNA transfection.

siRNA transfection was conducted by using Lipofectamine 3000 (L3000015; Thermo Fisher Scientific) in compliance with the protocol. Targeting sequences of double-stranded siRNAs were as follows: FXR-siRNA-1, 5′-GGACCATGAAGACCAGATT-3′; FXR-siRNA-2, 5′-GACCTCGACAACAAAGTCA-3′; HVEM-siRNA-1, 5′-GGCCTAATCATATGTGTGA-3′; HVEM-siRNA-2, 5′-GAGGAGACAATACCCTCAT-3′; negative control (NC), 5′-TTCTCCGAACGTGTCACGT-3′. All siRNA sequences were provided by GenePharma (Shanghai, China).

### Coculture assay.

FXR-overexpressed A549 cells transfected with HVEM-siRNAs or HVEM-overexpressed H1975 cells transfected with FXR-siRNAs were cultured in 48-well plates (2 × 10^4^ cells/well) overnight. Peripheral blood mononuclear cells (PBMCs) were separated by using Ficoll-Hypaque density gradient centrifugation (P8900; Solarbio). Then, CD8^+^ T cells were purified by EasySep Human CD8 Positive Selection Kit II (17853; Stemcell Technologies). The sorted human CD8^+^ T cells were activated by plate-bound anti-CD3 (4 μg/mL; 555329; BD Pharmingen), soluble anti-CD28 (1 μg/mL; 555725; BD Pharmingen), and IL-2 (5 ng/mL; 200-02; PeproTech, NJ) and were subsequently added to the cultured A549 or H1975 cells at a 25:1 effector-to-target cell ratio. For BTLA blockade assay, rat monoclonal anti–human BTLA antibody (5 μg/mL; AG-20B-0049; Adipogen) or isotype control rat IgG1 (5 μg/mL; AG-35B-0012PF; Adipogen) was added to cocultures of CD8^+^ T cells and A549 cells with or without FXR overexpression. After 3 days, the cocultured CD8^+^ T cells were harvested to stain with CD3-APC (17-0038-42; eBioscience), CD8-APC-eFluor 780 (47-0088-42; eBioscience), TNF-α–FITC (11-7349-81; eBioscience), and IFN-γ–PE (12-7319-41; eBioscience). Alternatively, they were stained with CD3-APC, CD8-APC-eFluor 780, and annexin V–FITC/PI (C1062M; Beyotime), and examined using flow cytometry. The supernatant was collected to detect the amount of released LDH by using LDH cytotoxicity testing kit (C0018M; Beyotime), complying with the recommended protocol. Finally, the cocultured A549 or H1975 cells were stained with crystal violet to determine cell viability.

### Flow cytometry.

Flow cytometry was conducted complying with our previous publication (16). The following fluorochrome-linked antibodies were utilized in compliance with the manufacturer’s recommendations: anti-human: HVEM-APC (318808; BioLegend, CA). Anti-mouse: CD45-FITC (103108; BioLegend), CD3-PerCP-Cyanine5.5 (100218; BioLegend), CD4-PE-Cyanine7 (100422; BioLegend), HVEM-PE (136304; BioLegend), PD-L1-APC (124311; BioLegend), NK1.1-APC (156506; BioLegend), BTLA-PE (134804; BioLegend), IFN-γ-PE (163503; BioLegend), Granzyme B-PE-Cyanine7 (372214; BioLegend), Foxp3-PE (126404; BioLegend), CD11b-PE-Cyanine7 (101215; BioLegend), CD11c-PerCP-Cyanine5.5 (117328; BioLegend), F4/80-PE (123110; BioLegend), CD206-APC (162506; BioLegend), CD8α-APC-Cyanine7 (A15386; ThermoFisher Scientific), Gr-1-APC-Cyanine7 (557661; BD Pharmingen), TNFα-eFlour 450 (48-7321-80; eBioscience), PD-1-eFlour 450 (48-9985-82; eBioscience), MHC-II-eFlour 450 (48-5321-80; eBioscience). For isotype controls, nonspecific mouse, rat, and hamster IgG were utilized at dilutions matched with the primary antibodies. At least 5 × 10^4^ events were collected from each sample for flow cytometry analysis.

### Luciferase reporter assay.

Luciferase reporter constructs harboring the WT or the nucleotide –3,537/–3,319 sequence-deleted promoter region of *TNFRSF14* gene were built by OBiO Biotechnology. Luciferase reporter experiments were conducted using Dual-Luciferase Reporter Assay System (E1960; Promega) in line with the recommended instructions. In brief, FXR-overexpressed A549 or FXR-silenced H1975 cells were cotransfected with a WT or the nucleotide –3,537/–3,319 sequence-deleted *TNFRSF14* promoter vector. A renilla luciferase reference vector was included in each transfection system. After 24 hours, luciferase activity was examined by using a luminometer (Promega) and normalized against corresponding renilla luciferase activity.

### ChIP assay.

ChIP was conducted using the SimpleChIP Plus Enzymatic Chromatin IP Kit (9005; Cell Signaling Technology). In brief, H1975, FXR-overexpressed A549, and FXR-silenced H1975 cells (1 × 10^7^) were cross-linked with 1% formaldehyde, quenched with 0.125 mol/L glycine, and lysed with SDS-containing buffer. The chromatin DNA was fragmented by partial digestion with Micrococcal Nuclease to obtain fragments of an average size of 100–900 bp, which was verified by electrophoresis on a 1% agarose gel and ethidium bromide staining. Then, the fragmented chromatin was aliquoted as input DNA or was immunoprecipitated with IgG or a mouse monoclonal anti–human FXR/*NR1H4* antibody (72105S; Cell Signaling Technology) at 4°C overnight. The immunocomplexes were pulled down with protein G magnetic beads (9006; Cell Signaling Technology), decrosslinked, and purified as described. The eluted samples and corresponding input DNA were analyzed by qPCR. Finally, the PCR products were separated by electrophoresis on a 1% agarose gel and visualized in the Image Lab Analysis System (Bio-Rad) The ChIP-qPCR primers used in this study are listed in Supplemental Table 1.

### Animal experiments.

For in vivo FXR inhibition study, 5-week-old female C57BL/6 mice were inoculated s.c. with 1 × 10^6^ LLC cells via right flank injection. When the tumors reached a volume of ~100 mm^3^, the mice were randomly grouped (random-number grouping method) and injected i.p. with increasing concentrations of UDCA (0, 50, 100, and 150 mg/kg, in 5% DMSO and 95% corn oil), DHP (0, 10, 20, and 40 mg/kg, in 10% DMSO and 90% corn oil), and Z-GS (0, 10, 20, and 40 mg/kg, in 10% DMSO and 90% corn oil) every 3 days for 15 days. For in vivo HVEM/BTLA blockade study, 5-week-old female C57BL/6 mice were inoculated s.c. with 1 × 10^6^ FXR-overexpressed or mock LLC cells via right flank injection. When the tumors reached a volume of ~100 mm^3^, the mice were randomly (random-number grouping method) assigned to the control and treatment groups, which were given i.p. with 200 μg InVivoMAb mouse IgG1 isotype control (BE0083; BioXCell) or 200 μg InVivoMAb anti-mouse BTLA/CD272 (BE0210; BioXCell) every 3 days, respectively. The tumor volume was estimated every 3 days and calculated as (length × width^2^)/2. The long-term survival of mice in each group was recorded. Another batch of C57BL/6 mice were randomly grouped (random-number grouping method) and treated with in vivo antibodies similarly. Mouse tumors were isolated when the tumor size reached ~1 cm^3^. In compliance with the recommended protocol, the mouse tumors were shredded and then digested by a Tumor Dissociation Kit (130-096-730; Miltenyi Biotec). Cell suspensions were filtered through a cell strainers (70 μm), and mouse lymphocytes were obtained by using the Mouse Lymphocyte Separation Medium (DKW33-R0100; Dakewe Biotech), which were subsequently prepared for flow cytometry analysis.

### Statistics.

Kolmogorov-Smirnov analysis was applied to test normality of the quantitative data. Bartlett’s and Levene’s tests were applied to examine homogeneity of variances. For 2-group comparisons, normally distributed data were examined via 2-tailed Student *t* test. The skewed distributed data were examined via Mann-Whitney *U* test. For mutiple-group comparisons, normally distributed data were examined by 1-way ANOVA, followed by Tukey’s post hoc test. The skewed distributed data were examined by Kruskal-Wallis rank sum test, followed by Dunnett’s post hoc test. In regards to categorical data, χ^2^ test was used for 2-group comparisons, and χ^2^ test followed by Bonferroni post hoc test was used for mutiple-group comparisons. Spearman’s rank correlation test was applied for correlation analysis. Survival curves were established by Kaplan-Meier method and examined by log-rank test. Data were analyzed by using SPSS v26.0 (IBM Corp.) and GraphPad Prism v8.0 (GraphPad Software). The *P* value (2-sided) < 0.05 was considered statistically significant.

### Study approval.

This study was conducted with the approval of the Ethics Committee of Shandong Provincial Hospital (Jinan, China, approval no. NSFC-2019-015). The research process was in accordance with relevant ethical principles of the Declaration of Helsinki. All participants have signed informed consent. All animal experimental procedures were in accordance with protocols approved by the IACUC in Shandong Provincial Hospital (Jinan, China, approval no. 2022-023).

### Data availability.

The *NR1H4* and *TNFRSF14* mRNA expression data in NSCLC generated in this study are publicly available in TCGA databases at https://portal.gdc.cancer.gov/ The TCGA accession codes are provided in Supplemental Table 2. Values used to generate graphs in figures and supplemental figures are available in the Supporting Data Values file.

## Author contributions

XX and BS contributed data curation, software, formal analysis, investigation, visualization, methodology and writing-original draft. HW, XJ, and JW contributed resources, methodology and visualization. JL and DL contributed software and validation. BL, XW, and LS contributed resources, formal analysis and validation. WY and SJ contributed conceptualization, data curation, supervision, funding acquisition, validation, project administration and writing-review and editing. All authors reviewed the manuscript.

## Supplementary Material

Supplemental data

Unedited blot and gel images

Supporting data values

## Version 1 09/23/2025

Electronic publication
