# Supplementary material for: FXR shapes an immunosuppressive microenvironment in PD-L1lo/– non-small cell lung cancer by upregulating HVEM
Source: JCI Insight. 2025 Sep 23;10(18):e190716. doi: 10.1172/jci.insight.190716 (PMC12487857; doi:10.1172/jci.insight.190716)
Supplement: Supplemental data [file jciinsight-10-190716-s200.pdf]

FXR shapes an immunosuppressive microenvironment in PD-L1$^{lo/-}$ non-small cell lung cancer by upregulating HVEM

Xiaolong Xu, Bin Shang, Hancheng Wu, Xiuye Jin, Junren Wang, Jing Li, Daowei Li, Bin Liang, Xingguang Wang, Lili Su, Wenjie You, and Shujuan Jiang

Supplementary figures and figure legends

Supplementary Figure 1.

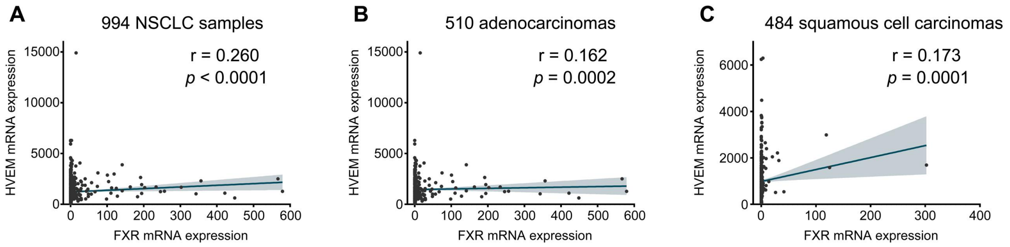

Supplementary Figure 1. FXR is positively correlated with the expression of HVEM in NSCLC from TCGA datasets. TCGA database were analyzed. Spearman's correlation test suggested that there was a significant positive correlation between the mRNA expression of FXR and HVEM either in 994 NSCLC (**A**, r = 0.260, $p < 0.0001$), or in 510 adenocarcinoma (**B**, r = 0.162, $p = 0.0002$), or in 484 squamous cell carcinoma samples (**C**, r = 0.173, $p = 0.0001$).

Supplementary Figure 2.

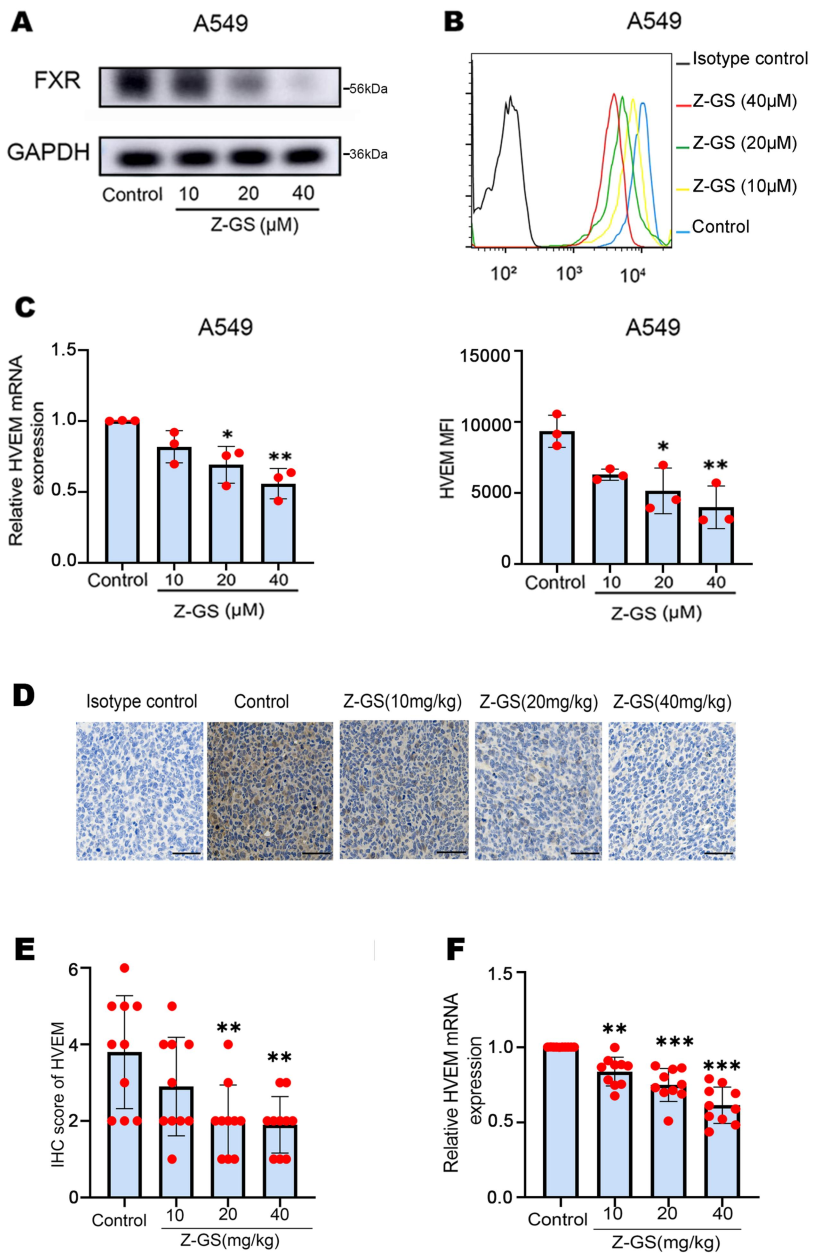

Supplementary Figure 2. Z-GS dose-dependently inhibit HVEM expression in NSCLC. A549 cells were treated with concentration gradients of Z-GS (0, 10, 20, and 40 μM) for 48 h. (**A**) The protein levels of FXR in A549 cells were examined by Western blotting. (**B**) Representative histograms and MFI quantifications for HVEM membrane staining in A549 cells were analyzed by flow cytometry. (**C**) Relative mRNA levels of HVEM in A549 cells were examined by q-PCR. β-Actin served as an internal control. C57BL/6 mice were inoculated s.c. with 1 × 10^6 LLC cells, and injected i.p. with increasing doses of Z-GS (0, 10, 20, and 40 mg/kg) every 3 days for 15 days when the tumors reached a volume of ~100 mm^3. Representative IHC images (**D,** magnification, ×200) and IHC score (**E**) of HVEM expression in mouse LLC

tumors of each group are shown. Non-specific rabbit IgG was used as an isotype control antibody. Scale bar represents 50 µm. (**F**) Relative mRNA levels of HVEM in mouse LLC tumors of each group were compared. Each experiment was conducted independently at least 3 times. Data are shown as mean ± SD from 3 biological replicates. For **D-F**, n = 10 mice/group. Statistical significance was assessed with 1-way ANOVA followed by Tukey's post hoc test (**B**, **C**, **F**) or Kruskal-Wallis rank sum test followed by Dunnett's post hoc test (**E**). * $p < 0.05$, ** $p < 0.01$, *** $p < 0.001$ compared with control group.

Supplementary Figure 3.

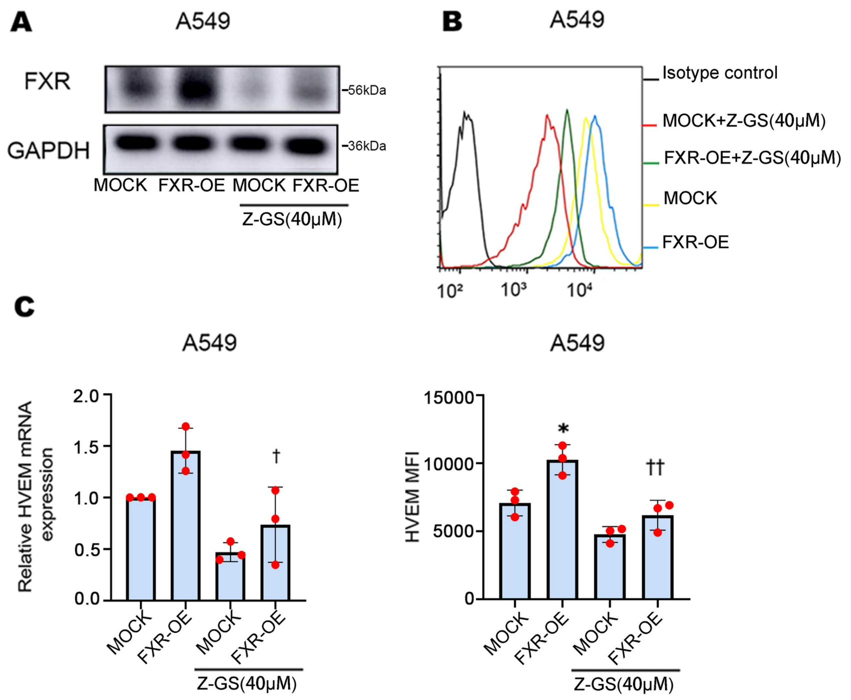

Supplementary Figure 3. FXR upregulates HVEM expression in NSCLC cells. FXR-overexpressed A549 stable cells were treated with 40 µM Z-GS for 48 h. (**A**) The protein levels of FXR were examined by Western blotting. (**B**) Representative histograms and MFI quantifications for HVEM membrane staining were analyzed by flow cytometry. (**C**) Relative mRNA levels of HVEM were examined by q-PCR. β-Actin served as an internal control. Each experiment was conducted independently at least 3 times. Data are shown as mean ± SD from 3 biological replicates. Statistical significance was assessed with 1-way ANOVA followed by Tukey's post hoc test (**B**, **C**). * $p < 0.05$, compared with mock group. † $p < 0.05$, †† $p < 0.01$, compared with control group. OE, overexpression.

Supplementary Figure 4.

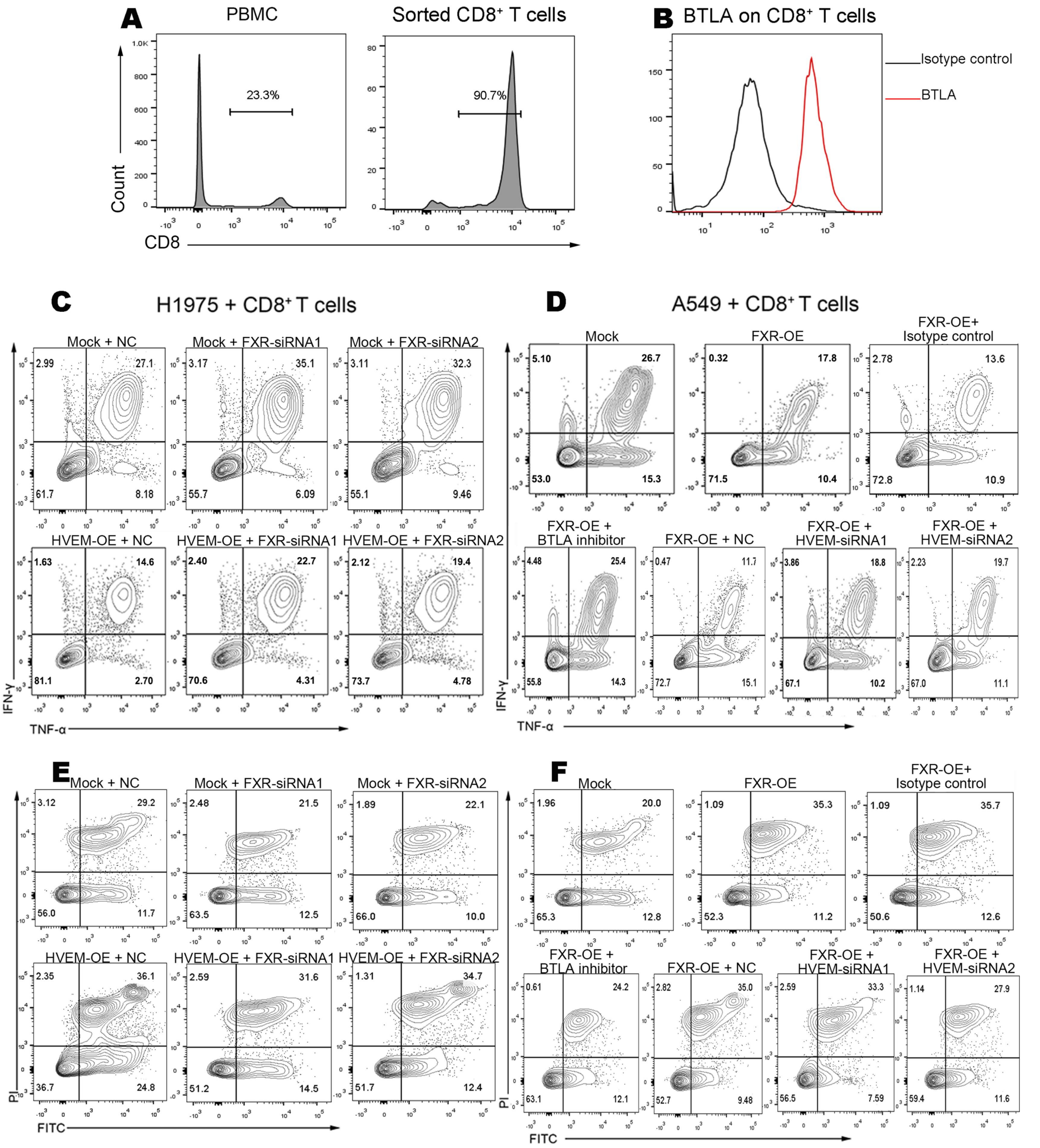

Supplementary Figure 4. FXR inhibits cytokine production and cytotoxic activity of cocultured CD8⁺ T cells through HVEM/BTLA checkpoint pathway. (**A**) The purity of sorted human CD8⁺ T cells in Figure 4 was examined by flow cytometry. (**B**) Representative flow cytometric histograms showing BTLA surface expression in sorted human CD8⁺ T cells in Figure 4. (**C-F**) Representative flow cytometric contour plots for Figure 4**C-G** and **I** are shown, respectively. Each experiment was conducted independently at least 3 times.

Supplementary Figure 5.

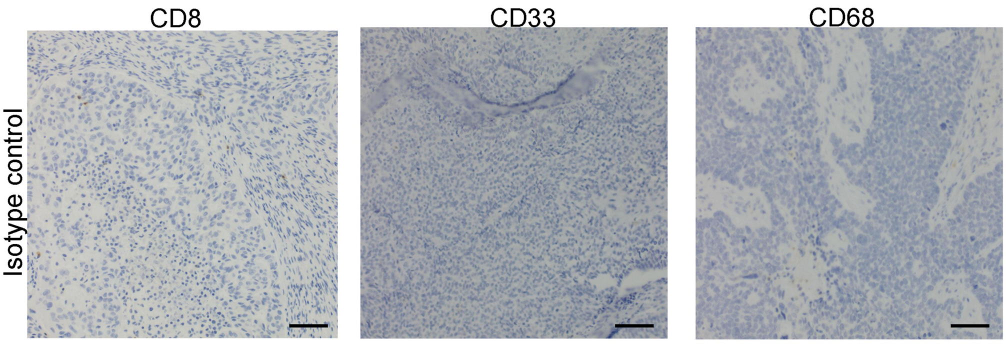

Supplementary Figure 5. Isotype control images for CD8, CD33 and CD68 staining in Figure 5A (magnification, ×80). Non-specific mouse or rabbit IgG was used as an isotype control antibody. Scale bar represents 50 µm.

Supplementary Figure 6.

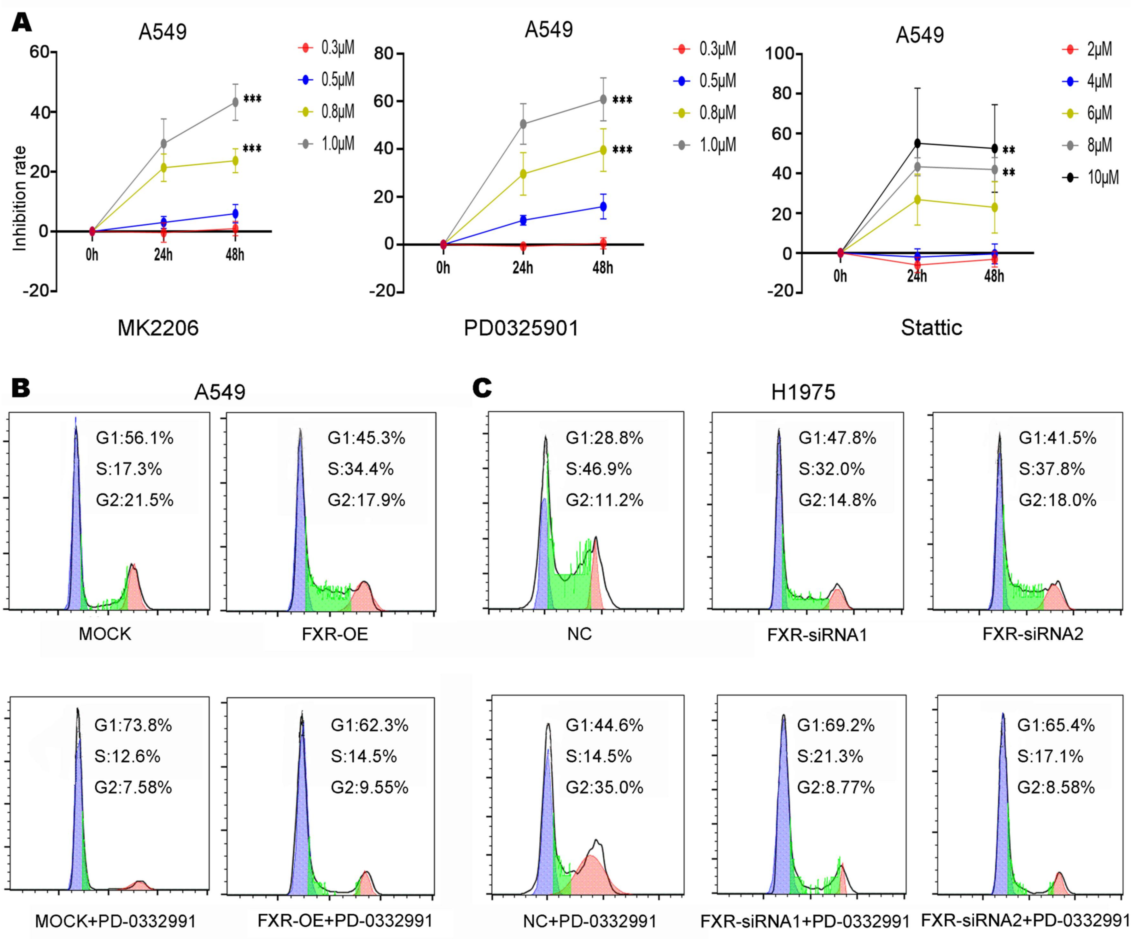

Supplementary Figure 6. FXR upregulates HVEM via transcriptional activation, intracellular Akt, Erk1/2 and STAT3 pathways, and cell cycle G1/S progression in NSCLC. (**A**) A549 cells were treated with increasing concentrations of MK2206 (0, 0.3, 0.5, 0.8 and 1 μM), PD0325901 (0, 0.3, 0.5, 0.8 and 1 μM) or Stattic (0, 2, 4, 6, 8 and 10 μM) for 24 and 48 h. The inhibition rate of cell proliferation was determined according to CCK-8 OD values, and the concentration and time effect curves were plotted. (**B** and **C**) Representative flow cytometric histograms showing the cell cycle distributions of A549 stable cells (**B**) and H1975 cells (**C**) in Figure 6**J** and **K**. Each experiment was conducted independently at least 3 times. Data are shown as mean ± SD from 3 biological replicates. Statistical significance was assessed with 1-way ANOVA followed by Tukey's post hoc test (**A**). ** $p < 0.01$, *** $p < 0.001$, compared with the control group.

Supplementary Figure 7.

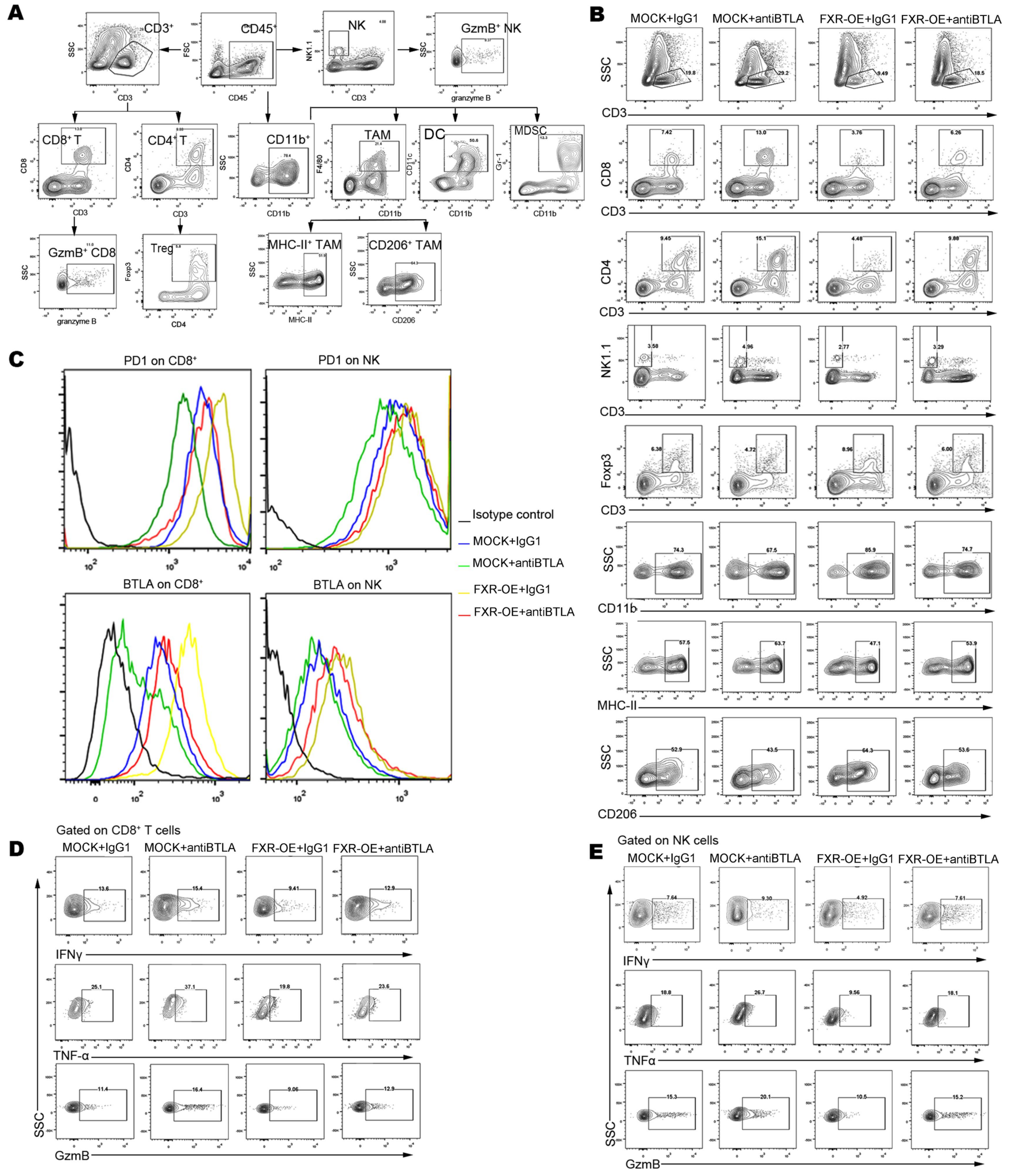

Supplementary Figure 7. HVEM/BTLA blockade immunotherapy reactivates TME and produces antitumor activity against FXR^{hi}PD-L1^{lo} mouse LLC tumors. (**A**) Gating strategy for flow cytometric analysis in Figure 7**E-G**, (**B-E**) Representative flow cytometric contour plots and histograms for Figure 7**E-G** are shown, respectively. Each experiment was conducted independently at least three times.

Supplementary tables

Supplementary Table 1. ChIP-qPCR primers used in this study.

Primer	Forward primer (5' to 3')	Reverse primer (5' to 3')
Primer 1	GTGAGGTGGGGAAACACAGAT	CAGGAGCTGTCCAGGCAAAG
Primer 2	ACCGCATTCTGGTGGTTTTAT	CTCACTCACACACTCAGGGAC
Primer 3	AGCAAGAGCTGCCCTTCCA	CTTCCCTTCCGAATAAAACCAC
Primer 4	TCCCTACCACCAGGCTCTGT	TGGAAGGGCAGCTCTTGCT
Primer 5	TGAAGTCAGGACCAGGAGGG	TGGAAGCCCAGGTCTGTCAC
Primer 6	CCTAACTTCACCCCTGCCTG	TGGCATCTGGTGGAAAGGAC
Primer 7	GCACCAGGGAAACAGCAGAT	AGGCAGGGGTGAAGTTAGG
Primer 8	GGGAGTGGAGGATGCAAGGG	AGACACGCAGGAGCATGGACT
Primer 9	CCTGTGTTAGTCTTGTCTCTTGCC	TCACCAACTCCCCAAGAACG
Primer 10	CAGGGCTTACTCAGCAATCAGA	AAGAGACAAGACTAACACAGGCATC
Primer 11	AGCCCACGCTGAATGGATGA	GTCCCTTTTCTCCCTTCTCTGT
Primer 12	GCAGCCGTGTCAATGCAGAT	ACTCCTTCATCCATTCAGCGT
Primer 13	CCGCAGTTTCCTCTTCATCC	CAGACCCAATGCTGTGCCA
Primer 14	CTCACGCCTCCATCTGCTCT	AGTGTGTGAGTTGAGTGCTGCT
Primer 15	GACACAGAGCCCTCCAAGTTC	AGAGCAGATGGAGGCGTGAG
Primer 16	AGGCACTTGAGACAGAGGCA	GTGGTCAGAGCATTGTGGGA
Primer 17	GGAGGAGAGGGTCTGAGGAAT	GTCTTTCTGACTCGGGGATGG
Primer 18	AGGTGGGGATGTTGAAGGTG	TCCTCAGACCCTCTCCTCCA
Primer 19	CCATGTATTGAACCAGGACGG	CCTTCAACATCCCCACCTTC
Primer 20	TCAGCCGTTGCATGTTCCT	ACCTCCCGTCCTGGTTCAAT
Primer for FXR-binding site in human *SHP* promoter	GTGATAAGGCACTTCCAGGTTG	GATGACTCAAGTGGATAAACAAGGT

Supplementary Table 2. lusc_tcga_pan_can_atlas_2018.

SAMPLE_ID	NR1H4	TNFRSF14	SAMPLE_ID	NR1H4	TNFRSF14
TCGA-95-8494-01	0	2416.92	TCGA-97-8172-01	1.0585	1703.05
TCGA-95-8039-01	0	1519.15	TCGA-97-8171-01	0	1957.46
TCGA-95-7948-01	0	1537.57	TCGA-97-7941-01	10.9977	3079.53
TCGA-95-7947-01	0.3056	809.224	TCGA-97-7938-01	1.2085	679.565
TCGA-95-7944-01	1.8302	2070.72	TCGA-97-7937-01	0	768.767
TCGA-95-7567-01	0.2813	905.134	TCGA-97-7554-01	0	706.758
TCGA-95-7562-01	0	746.055	TCGA-97-7553-01	2.6788	1140.09
TCGA-95-7043-01	6.2708	923.3	TCGA-97-7552-01	5.1154	1638.09
TCGA-95-7039-01	0.7143	1112.01	TCGA-97-7547-01	0.4377	883.16
TCGA-93-A4JQ-01	1.3078	2161.3	TCGA-97-7546-01	0	772.949
TCGA-93-A4JP-01	4.7696	1615.69	TCGA-95-A4VP-01	2.6209	2223.51
TCGA-93-A4JO-01	1.046	3503.79	TCGA-95-A4VN-01	0	1699.18
TCGA-93-A4JN-01	0	2289.59	TCGA-95-A4VK-01	47.3402	1491.36
TCGA-93-8067-01	9.6845	1182.27	TCGA-92-7341-01	0	297.878
TCGA-93-7348-01	0.6604	1673	TCGA-92-7340-01	0	510.662
TCGA-93-7347-01	2.331	1714.42	TCGA-90-A59Q-01	0.6116	3769.11
TCGA-91-A4BD-01	0	2136.05	TCGA-90-A4EE-01	0.4523	2532.3
TCGA-91-A4BC-01	0	2522.71	TCGA-90-A4ED-01	0	1055.66
TCGA-91-8499-01	0	711.989	TCGA-90-7964-01	0	1256.89
TCGA-91-8497-01	4.6335	2545.46	TCGA-90-7769-01	0	70.6065
TCGA-91-8496-01	2.3232	2081.27	TCGA-90-7767-01	0	880.168
TCGA-91-7771-01	28.5714	1555.97	TCGA-90-7766-01	0	894.319
TCGA-91-6849-01	3.337	802.403	TCGA-90-6837-01	0.4153	1164.93
TCGA-91-6848-01	0	651.973	TCGA-85-A5B5-01	0	1176.06
TCGA-91-6847-01	2.0562	311.81	TCGA-85-A53L-01	0	997.672
TCGA-91-6840-01	48.9009	1008.44	TCGA-85-A513-01	0.6452	3733.22
TCGA-91-6836-01	0	603.039	TCGA-85-A512-01	0	840.825
TCGA-91-6835-01	0.4515	1525.55	TCGA-85-A511-01	0	1307.45
TCGA-91-6831-01	0	567.201	TCGA-85-A510-01	0.5528	747.363
TCGA-91-6830-01	25.2874	870.621	TCGA-85-A50Z-01	0	830.907
TCGA-91-6829-01	20.1342	318.327	TCGA-85-A50M-01	0	593.181
TCGA-91-6828-01	0	863.519	TCGA-85-A4QR-01	0	434.977
TCGA-86-A4P8-01	3.6463	3996.02	TCGA-85-A4QQ-01	0	315.507
TCGA-86-A4P7-01	0.9488	2732	TCGA-85-A4PA-01	2.8253	6297.95
TCGA-86-A4JF-01	0	1332.03	TCGA-85-A4JC-01	0	1162.94
TCGA-86-A4D0-01	0	479.828	TCGA-85-A4JB-01	0	921.895
TCGA-86-A456-01	2.3658	2301.64	TCGA-85-A4CN-01	0	803.195
TCGA-86-8674-01	0	595.11	TCGA-85-A4CL-01	0	1051.03
TCGA-86-8673-01	0	1688.52	TCGA-85-8666-01	0	503.349
TCGA-86-8672-01	0	1374.94	TCGA-85-8664-01	0.5113	620.279
TCGA-86-8671-01	1.6531	4066.39	TCGA-85-8584-01	0	2262.2
TCGA-86-8669-01	3.1506	620.176	TCGA-85-8582-01	0.657	968.035

TCGA-86-8668-01	0	1923.57	TCGA-85-8580-01	0	560.147
TCGA-86-8585-01	39.7014	2507.35	TCGA-85-8481-01	0	1290.01
TCGA-86-8359-01	194.411	1949.54	TCGA-85-8479-01	0	253.143
TCGA-86-8358-01	0	926.84	TCGA-85-8355-01	4.6394	1629.36
TCGA-86-8281-01	0.6964	684.391	TCGA-85-8354-01	0	565.158
TCGA-86-8280-01	0.3635	2450.52	TCGA-85-8353-01	0.4413	880.591
TCGA-86-8279-01	0	935.07	TCGA-85-8352-01	0	376.043
TCGA-86-8278-01	0	1613.89	TCGA-85-8351-01	0	394.09
TCGA-86-8076-01	0.7796	1501.23	TCGA-85-8350-01	2.8737	1596.05
TCGA-86-8075-01	13.4267	970.678	TCGA-85-8288-01	1.6105	1600.87
TCGA-86-8074-01	1.4296	1075.84	TCGA-85-8287-01	0	1224.32
TCGA-86-8073-01	3.0933	1210.15	TCGA-85-8277-01	0.4409	769.758
TCGA-86-8056-01	191.235	2646.4	TCGA-85-8276-01	0	1049.51
TCGA-86-8055-01	5.5722	733.733	TCGA-85-8072-01	0	1235.88
TCGA-86-8054-01	0.333	246.894	TCGA-85-8071-01	0	482.84
TCGA-86-7955-01	0.6025	236.289	TCGA-85-8070-01	0	182.386
TCGA-86-7954-01	202.571	1641.86	TCGA-85-8052-01	0	547.059
TCGA-86-7953-01	0	1947.58	TCGA-85-8049-01	0.52	1248.89
TCGA-86-7714-01	0	1041.05	TCGA-85-8048-01	0	1688.01
TCGA-86-7713-01	1.4429	529.74	TCGA-85-7950-01	0.4903	1912.19
TCGA-86-7711-01	0	413.543	TCGA-85-7844-01	0	898.34
TCGA-86-7701-01	1.0537	1052.28	TCGA-85-7843-01	0	809.816
TCGA-86-6851-01	0.8545	2891.57	TCGA-85-7710-01	0.3423	748.119
TCGA-86-6562-01	0.536	839.609	TCGA-85-7699-01	0.5519	889.71
TCGA-83-5908-01	0	1715.21	TCGA-85-7698-01	0	667.901
TCGA-80-5611-01	0.5574	2693.57	TCGA-85-7697-01	0.2862	821.928
TCGA-80-5608-01	4.6104	1328.6	TCGA-85-7696-01	0	520.688
TCGA-80-5607-01	112.286	1616.55	TCGA-85-6798-01	0	184.33
TCGA-78-8662-01	0	184.586	TCGA-85-6561-01	0.2793	724.822
TCGA-78-8660-01	0	1895.64	TCGA-85-6560-01	0	442.32
TCGA-78-8655-01	1.2666	1567.17	TCGA-85-6175-01	1.6854	767.275
TCGA-78-8648-01	0	2374.16	TCGA-79-5596-01	0.6879	280.409
TCGA-78-8640-01	0	858.287	TCGA-77-A5GH-01	0	3217.73
TCGA-78-7633-01	3.167	547.305	TCGA-77-A5GF-01	0	437.132
TCGA-78-7542-01	0	968.885	TCGA-77-A5GB-01	0	1013.92
TCGA-78-7540-01	3.9768	1844.88	TCGA-77-A5G8-01	0	609.56
TCGA-78-7539-01	0.5891	3800.8	TCGA-77-A5G7-01	0	576.575
TCGA-78-7537-01	0.7133	1313.05	TCGA-77-A5G6-01	0	658.074
TCGA-78-7536-01	0	470.833	TCGA-77-A5G3-01	0	501.608
TCGA-78-7535-01	0	2047.09	TCGA-77-A5G1-01	0	1028.17
TCGA-78-7220-01	183.245	755.584	TCGA-77-A5FZ-01	0.597	2744.94
TCGA-78-7167-01	0.8915	1023.12	TCGA-77-8156-01	0	1108.56
TCGA-78-7166-01	18.7249	1474.99	TCGA-77-8154-01	0	642.393
TCGA-78-7163-01	0.3424	835.984	TCGA-77-8153-01	0	232.314

TCGA-78-7162-01	1.4636	804.775	TCGA-77-8150-01	0	834.994
TCGA-78-7161-01	0.3674	807.632	TCGA-77-8148-01	0	576.459
TCGA-78-7160-01	48.5804	785.584	TCGA-77-8145-01	0	1118.39
TCGA-78-7159-01	0	586.623	TCGA-77-8144-01	0	205.227
TCGA-78-7158-01	0	854.556	TCGA-77-8143-01	41.2889	541.458
TCGA-78-7156-01	11.194	757.413	TCGA-77-8140-01	0	2046.7
TCGA-78-7155-01	0	87.3148	TCGA-77-8139-01	0.6207	454.904
TCGA-78-7154-01	1.786	541.98	TCGA-77-8138-01	0	1191.56
TCGA-78-7153-01	9.825	1028.75	TCGA-77-8136-01	0	1603.46
TCGA-78-7152-01	0.9285	974.392	TCGA-77-8133-01	1.087	1386.78
TCGA-78-7150-01	25.8176	478.296	TCGA-77-8131-01	0.6925	2537.83
TCGA-78-7149-01	5.5142	1244.03	TCGA-77-8130-01	0	358.885
TCGA-78-7148-01	9.5719	522.356	TCGA-77-8128-01	0	299.177
TCGA-78-7147-01	7.825	1535.04	TCGA-77-8009-01	0	375.025
TCGA-78-7146-01	0	486.144	TCGA-77-8008-01	0	1144.06
TCGA-78-7145-01	0	383.677	TCGA-77-8007-01	0	1378.66
TCGA-78-7143-01	0.4798	1270.05	TCGA-77-7465-01	1.2054	770.788
TCGA-75-7031-01	13.9785	786.624	TCGA-77-7463-01	0	291.862
TCGA-75-7030-01	3.125	1367.2	TCGA-77-7338-01	0	304.687
TCGA-75-7027-01	0	556.737	TCGA-77-7337-01	0	482.894
TCGA-75-7025-01	2.6475	2881.8	TCGA-77-7335-01	0	988.343
TCGA-75-6214-01	12.4516	731.386	TCGA-77-7142-01	0.369	269.342
TCGA-75-6212-01	0	1349.96	TCGA-77-7141-01	0	275.056
TCGA-75-6211-01	0	719.31	TCGA-77-7140-01	0.3746	120.993
TCGA-75-6207-01	0	439.332	TCGA-77-7139-01	0	286.743
TCGA-75-6206-01	0	1124.2	TCGA-77-7138-01	0	299.181
TCGA-75-6205-01	0.8475	1154.72	TCGA-77-6845-01	0	181.073
TCGA-75-6203-01	2.0633	1592.71	TCGA-77-6844-01	0	1037.66
TCGA-75-5147-01	110.292	1107.67	TCGA-77-6843-01	0.4439	147.208
TCGA-75-5146-01	4.2523	934.004	TCGA-77-6842-01	0.8576	3531.66
TCGA-75-5126-01	2.3885	1255.88	TCGA-70-6723-01	0	335.935
TCGA-75-5125-01	0	1008.31	TCGA-70-6722-01	0	591.162
TCGA-75-5122-01	0	2060.26	TCGA-6A-AB49-01	0	2871.59
TCGA-73-A9RS-01	0	633.785	TCGA-68-A59J-01	0	1372.03
TCGA-73-7499-01	3.581	1435.93	TCGA-68-A59I-01	0	857.639
TCGA-73-7498-01	0.5451	1495.07	TCGA-68-8251-01	0	949.048
TCGA-73-4677-01	0.2891	532.403	TCGA-68-8250-01	0	913.642
TCGA-73-4676-01	2.1108	498.343	TCGA-68-7757-01	0	1298.86
TCGA-73-4675-01	0	1704.51	TCGA-68-7756-01	0.3096	682.774
TCGA-73-4670-01	0	746.549	TCGA-68-7755-01	0.3087	318.614
TCGA-73-4668-01	0	1075.78	TCGA-66-2800-01	0	564.813
TCGA-73-4666-01	0.2515	1268.47	TCGA-66-2795-01	0	519.333
TCGA-73-4662-01	0.7033	922.74	TCGA-66-2794-01	0	1136.76
TCGA-73-4659-01	0.6329	1056.39	TCGA-66-2793-01	0	280.313

TCGA-73-4658-01	0	785	TCGA-66-2792-01	0	407.656
TCGA-71-8520-01	0.62	1942.44	TCGA-66-2791-01	0	1091.4
TCGA-71-6725-01	0	796.124	TCGA-66-2790-01	8.2361	1549.31
TCGA-69-A59K-01	0	1889.41	TCGA-66-2789-01	2.9884	818.648
TCGA-69-8453-01	1.462	2209.02	TCGA-66-2788-01	0	886.566
TCGA-69-8255-01	194.175	1081.9	TCGA-66-2787-01	0	546.207
TCGA-69-8254-01	104.091	2245.49	TCGA-66-2786-01	0	1281
TCGA-69-8253-01	0.8571	853.3	TCGA-66-2785-01	0	1956.9
TCGA-69-7980-01	1.2248	1148.67	TCGA-66-2783-01	0	705.486
TCGA-69-7979-01	0	4580.45	TCGA-66-2782-01	0	1305.58
TCGA-69-7978-01	0	1594.16	TCGA-66-2781-01	0	644.101
TCGA-69-7974-01	0	976.875	TCGA-66-2780-01	0.3646	1203.4
TCGA-69-7973-01	0.258	635.089	TCGA-66-2778-01	0.4202	731.958
TCGA-69-7765-01	0.6242	684.875	TCGA-66-2777-01	0	580.192
TCGA-69-7764-01	15.9151	839.735	TCGA-66-2773-01	0	1102.29
TCGA-69-7763-01	1.1822	749.786	TCGA-66-2771-01	1.1655	1348.91
TCGA-69-7761-01	3.238	1852.97	TCGA-66-2770-01	0.6761	1137.34
TCGA-69-7760-01	0	461.387	TCGA-66-2769-01	0.3784	904.287
TCGA-67-6217-01	0.5686	1773.6	TCGA-66-2768-01	0	1541.17
TCGA-67-6216-01	0	1612.51	TCGA-66-2767-01	1.1355	1948.29
TCGA-67-6215-01	0	763.972	TCGA-66-2766-01	0	412.979
TCGA-67-4679-01	1.7214	1695.68	TCGA-66-2765-01	0	820.041
TCGA-67-3774-01	0	1048.86	TCGA-66-2763-01	0	833.63
TCGA-67-3773-01	1.0277	1894.09	TCGA-66-2759-01	0	742.015
TCGA-67-3772-01	1.8727	1349.48	TCGA-66-2758-01	0	1049.34
TCGA-67-3771-01	2.6059	1643.79	TCGA-66-2757-01	0.7449	682.164
TCGA-67-3770-01	1.8797	2127.86	TCGA-66-2756-01	0	341.091
TCGA-64-5815-01	0.7855	719.159	TCGA-66-2755-01	0	2190.16
TCGA-64-5781-01	1.2862	2409.97	TCGA-66-2754-01	1.8174	357.36
TCGA-64-5779-01	0	984.853	TCGA-66-2753-01	0.6745	326.919
TCGA-64-5778-01	0	1622.32	TCGA-66-2744-01	0.4602	1136.6
TCGA-64-5775-01	0.4641	219.918	TCGA-66-2742-01	0.9413	715.425
TCGA-64-5774-01	0	745.976	TCGA-66-2737-01	0	750.327
TCGA-64-1681-01	1.8072	1465	TCGA-66-2734-01	0	987.724
TCGA-64-1680-01	0	1100.28	TCGA-66-2727-01	0	746.104
TCGA-64-1679-01	0	527.995	TCGA-63-A5MY-01	0	110.526
TCGA-64-1678-01	0	301.363	TCGA-63-A5MW-01	0	1202.33
TCGA-64-1677-01	1.0941	1248.06	TCGA-63-A5MV-01	0	1667.63
TCGA-64-1676-01	0.6929	1155.42	TCGA-63-A5MU-01	0	704.361
TCGA-62-A472-01	0	1283.23	TCGA-63-A5MT-01	0	3825.29
TCGA-62-A471-01	342.711	1039.43	TCGA-63-A5MS-01	0	2456.5
TCGA-62-A470-01	0	724.212	TCGA-63-A5MR-01	0	832.062
TCGA-62-A46Y-01	0.5013	1543.52	TCGA-63-A5MP-01	0.5122	1017.4
TCGA-62-A46V-01	0	932.784	TCGA-63-A5MN-01	0.5163	1944.7

TCGA-62-A46U-01	14.3855	2562.22	TCGA-63-A5MM-01	0	2135.12
TCGA-62-A46S-01	21.6336	1362.79	TCGA-63-A5ML-01	0	590.187
TCGA-62-A46R-01	1.5007	1372.05	TCGA-63-A5MJ-01	0.627	320.053
TCGA-62-A46P-01	25.5639	4071.17	TCGA-63-A5MI-01	0	225.492
TCGA-62-A46O-01	1.6722	140.084	TCGA-63-A5MH-01	0	905.34
TCGA-62-8402-01	0	2895.84	TCGA-63-A5MG-01	0	1298.99
TCGA-62-8399-01	2.6475	416.422	TCGA-63-A5MB-01	0.4321	669.06
TCGA-62-8398-01	141.123	1333.09	TCGA-63-A5M9-01	0	503.873
TCGA-62-8397-01	0	3143.62	TCGA-63-7023-01	0	482.554
TCGA-62-8395-01	0.8772	1603.1	TCGA-63-7022-01	0	1364.21
TCGA-62-8394-01	10.3961	1502.83	TCGA-63-7021-01	0	1461.65
TCGA-55-A57B-01	0.7052	2088.51	TCGA-63-7020-01	0.639	2536.37
TCGA-55-A4DG-01	0	2448.66	TCGA-63-6202-01	0	947.358
TCGA-55-A4DF-01	0.4623	1161.14	TCGA-63-5131-01	0.4028	979.365
TCGA-55-A494-01	0.4435	687.027	TCGA-63-5128-01	1.096	278.241
TCGA-55-A493-01	0.4848	2111.35	TCGA-60-2726-01	3.8802	2017.57
TCGA-55-A492-01	11.7836	1951.15	TCGA-60-2725-01	0	1261.79
TCGA-55-A491-01	13.7639	1181.88	TCGA-60-2724-01	0	571.226
TCGA-55-A490-01	0	279.887	TCGA-60-2723-01	0	650.494
TCGA-55-A48Z-01	11.0524	2692.26	TCGA-60-2722-01	0	179.195
TCGA-55-A48Y-01	28.8866	590.01	TCGA-60-2721-01	0	1770.95
TCGA-55-A48X-01	0.9775	2100.53	TCGA-60-2720-01	0	1455.82
TCGA-55-8621-01	1.6071	2022.42	TCGA-60-2719-01	0.3033	381.248
TCGA-55-8620-01	0	1437.15	TCGA-60-2716-01	0	1201.89
TCGA-55-8619-01	4.0769	2596.1	TCGA-60-2714-01	0	1027.83
TCGA-55-8616-01	0	1047.68	TCGA-60-2713-01	0	968.684
TCGA-55-8615-01	0	867.973	TCGA-60-2711-01	0	733.417
TCGA-55-8614-01	0	2002.06	TCGA-60-2710-01	0.6357	683.817
TCGA-55-8514-01	0.6452	2555.94	TCGA-60-2709-01	1.1475	1324
TCGA-55-8513-01	2.2255	3690.28	TCGA-60-2708-01	0	417.632
TCGA-55-8512-01	3.7413	2136.2	TCGA-60-2707-01	0.3659	705.884
TCGA-55-8511-01	0	1386.28	TCGA-60-2706-01	0.6835	666.551
TCGA-55-8510-01	0	3042.23	TCGA-60-2704-01	0.3761	803.689
TCGA-55-8508-01	0	1106.4	TCGA-60-2703-01	0	527.773
TCGA-55-8507-01	0	955.622	TCGA-60-2698-01	0.3531	863.305
TCGA-55-8506-01	0	1350.47	TCGA-60-2697-01	1.4421	1816.99
TCGA-55-8505-01	100.766	2877.73	TCGA-60-2695-01	0	856.064
TCGA-55-8302-01	0	1958.77	TCGA-58-A46N-01	0	343.298
TCGA-55-8301-01	1.2903	2226.33	TCGA-58-A46M-01	0	575.316
TCGA-55-8299-01	1.2671	1539.01	TCGA-58-A46L-01	0.9072	682.552
TCGA-55-8208-01	0.5157	2147.25	TCGA-58-A46K-01	0	666.171
TCGA-55-8207-01	0.7265	1054.81	TCGA-58-A46J-01	1.2346	1093.81
TCGA-55-8206-01	1.1813	1680.47	TCGA-58-8393-01	0	1562.43
TCGA-55-8205-01	0	1312.88	TCGA-58-8392-01	0	361.853

TCGA-55-8204-01	0.5365	777.28	TCGA-58-8391-01	0	534.51
TCGA-55-8203-01	0.4044	1110.22	TCGA-58-8390-01	0	1231.18
TCGA-55-8097-01	3.2026	1583.41	TCGA-58-8388-01	0.5161	2136.48
TCGA-55-8096-01	12.474	1926.19	TCGA-58-8387-01	0	1361.19
TCGA-55-8094-01	1.1921	259.288	TCGA-58-8386-01	0	1056.03
TCGA-55-8092-01	0	2475.95	TCGA-56-A62T-01	0.38	719.692
TCGA-55-8091-01	0	1881	TCGA-56-A5DS-01	0	1346.07
TCGA-55-8090-01	0	1671.06	TCGA-56-A5DR-01	0.8881	601.847
TCGA-55-8089-01	0	1903.42	TCGA-56-A4ZK-01	0	1112.45
TCGA-55-8087-01	0	1633.08	TCGA-56-A4ZJ-01	0.447	1803.37
TCGA-55-8085-01	1.0438	1263.87	TCGA-56-A4BY-01	0	1016.29
TCGA-55-7995-01	0	2099.75	TCGA-56-A4BX-01	0	333.751
TCGA-55-7994-01	0	1522.99	TCGA-56-A4BW-01	0	6243.94
TCGA-55-7914-01	0.4998	735.087	TCGA-56-A49D-01	0.501	1382.16
TCGA-55-7913-01	0	1396.5	TCGA-56-8629-01	0	1627.17
TCGA-55-7911-01	0	904.29	TCGA-56-8628-01	29.48	2212.97
TCGA-55-7910-01	0	835.954	TCGA-56-8626-01	0	1689.13
TCGA-55-7907-01	0	861.994	TCGA-56-8625-01	1.0499	1661.92
TCGA-55-7903-01	0	1348.8	TCGA-56-8624-01	0	1224.62
TCGA-55-7816-01	0	1239.33	TCGA-56-8623-01	7.169	2651.54
TCGA-55-7815-01	0	169.95	TCGA-56-8622-01	0.5047	681.45
TCGA-55-7728-01	0	5940.57	TCGA-56-8504-01	0	754.011
TCGA-55-7727-01	1.8349	362.752	TCGA-56-8503-01	0.6452	1499.3
TCGA-55-7726-01	0	334.809	TCGA-56-8309-01	0.8562	3506.45
TCGA-55-7725-01	4.2017	629.758	TCGA-56-8308-01	0	710.35
TCGA-55-7724-01	0	232.054	TCGA-56-8307-01	0	2780.55
TCGA-55-7576-01	0.4888	814.875	TCGA-56-8305-01	0	576.181
TCGA-55-7574-01	0.7938	2061.24	TCGA-56-8304-01	0.5565	568.188
TCGA-55-7573-01	0.8006	1685.57	TCGA-56-8201-01	0	1393.93
TCGA-55-7570-01	0	305.603	TCGA-56-8083-01	0	611.625
TCGA-55-7284-01	2.4933	1517.23	TCGA-56-8082-01	0	705.99
TCGA-55-7283-01	0	701.564	TCGA-56-7823-01	0	198.63
TCGA-55-7281-01	0	1977.48	TCGA-56-7822-01	0	656.567
TCGA-55-7227-01	0	1396.36	TCGA-56-7731-01	0	921.779
TCGA-55-6987-01	0.7062	1660.18	TCGA-56-7730-01	0	466.964
TCGA-55-6986-01	0.7267	804.731	TCGA-56-7582-01	0.5166	337.166
TCGA-55-6985-01	0	1031	TCGA-56-7580-01	0	846.701
TCGA-55-6984-01	0	2364.71	TCGA-56-7579-01	0	502.078
TCGA-55-6983-01	0.3546	1252.38	TCGA-56-7223-01	0	147.641
TCGA-55-6982-01	3.6875	1576.7	TCGA-56-7222-01	0	491.335
TCGA-55-6981-01	0	1308.71	TCGA-56-7221-01	0	294.207
TCGA-55-6980-01	1.7683	1346.38	TCGA-56-6546-01	13.8602	979.508
TCGA-55-6979-01	0.7047	1852.9	TCGA-56-6545-01	0.2772	648.218
TCGA-55-6978-01	2.5218	1250.73	TCGA-56-5898-01	0.4373	799.964

TCGA-55-6975-01	0	300.595	TCGA-56-5897-01	0	864.433
TCGA-55-6972-01	0	668.014	TCGA-56-1622-01	0	232.701
TCGA-55-6971-01	0	1520.47	TCGA-52-7812-01	0.3997	932.83
TCGA-55-6970-01	17.6678	617.944	TCGA-52-7811-01	0	504.48
TCGA-55-6969-01	0.3449	1034.86	TCGA-52-7810-01	0	306.28
TCGA-55-6968-01	0	1061.16	TCGA-52-7809-01	0.3453	1038.65
TCGA-55-6712-01	0	1675.69	TCGA-52-7622-01	0	946.424
TCGA-55-6642-01	0	693.486	TCGA-51-4081-01	0	353.197
TCGA-55-6543-01	0	1216.53	TCGA-51-4080-01	0	245.684
TCGA-55-5899-01	0.5715	762.732	TCGA-51-4079-01	0.4015	395.005
TCGA-55-1596-01	0	696.205	TCGA-46-6026-01	0	290.835
TCGA-55-1595-01	0	983.687	TCGA-46-6025-01	0.2526	331.937
TCGA-55-1594-01	0.3931	439.858	TCGA-46-3769-01	0.705	1006.86
TCGA-55-1592-01	0.8119	2056.98	TCGA-46-3768-01	0	321.079
TCGA-53-A4EZ-01	0.7869	3123.23	TCGA-46-3767-01	0.5066	848.775
TCGA-53-7813-01	2.0555	377.873	TCGA-46-3766-01	1.2862	1135.25
TCGA-53-7626-01	0.5691	1239.52	TCGA-46-3765-01	0	432.301
TCGA-53-7624-01	0.8783	558.586	TCGA-43-A56V-01	0.5155	922.475
TCGA-50-8460-01	0.6196	1783.1	TCGA-43-A56U-01	0.6286	2748.16
TCGA-50-8459-01	1.119	1921.47	TCGA-43-A475-01	0	2195.43
TCGA-50-8457-01	2.2193	1443.5	TCGA-43-A474-01	0	277.022
TCGA-50-7109-01	0	682.045	TCGA-43-8118-01	8.7638	809.447
TCGA-50-6673-01	98.1492	523.247	TCGA-43-8116-01	0	1433.07
TCGA-50-6597-01	16.0226	1556.01	TCGA-43-8115-01	0	1155.24
TCGA-50-6595-01	0	687.678	TCGA-43-7658-01	0	657.638
TCGA-50-6594-01	0	983.423	TCGA-43-7657-01	0	529.567
TCGA-50-6593-01	0	923.188	TCGA-43-7656-01	0	1261.69
TCGA-50-6592-01	0	1315.55	TCGA-43-6773-01	0.5478	1186.6
TCGA-50-6591-01	0	184.869	TCGA-43-6771-01	0	536.489
TCGA-50-6590-01	0	811.087	TCGA-43-6770-01	0	583.379
TCGA-50-5946-01	10.936	1111.48	TCGA-43-6647-01	0.3744	576.28
TCGA-50-5944-01	0	922.574	TCGA-43-6143-01	0	474.905
TCGA-50-5942-01	1.2786	819.34	TCGA-43-5670-01	0.3586	1018.74
TCGA-50-5941-01	0	1207.21	TCGA-43-5668-01	0	1120.44
TCGA-50-5939-01	8.2093	872.146	TCGA-43-3920-01	2.7425	568.379
TCGA-50-5936-01	29.2398	470.168	TCGA-43-3394-01	0.382	506.236
TCGA-50-5935-01	0	866.707	TCGA-43-2581-01	0	1034.28
TCGA-50-5933-01	0	566.846	TCGA-43-2578-01	0	625.273
TCGA-50-5932-01	0	392.436	TCGA-43-2576-01	0	1232.01
TCGA-50-5931-01	0	453.775	TCGA-39-5040-01	0.6207	342.107
TCGA-50-5930-01	0	743.404	TCGA-39-5039-01	2.0134	1057.5
TCGA-50-5072-01	18.0139	530.259	TCGA-39-5037-01	0.4172	277.322
TCGA-50-5068-01	0.8551	2213.23	TCGA-39-5036-01	0	729.23
TCGA-50-5066-01	0	1780.74	TCGA-39-5035-01	0	1155.69

TCGA-50-5055-01	11.6279	2185.41	TCGA-39-5034-01	0.5995	397.44
TCGA-50-5051-01	0	834.973	TCGA-39-5031-01	0	818.111
TCGA-50-5049-01	0.558	1773.42	TCGA-39-5030-01	0	1523.17
TCGA-50-5045-01	1.269	1987.59	TCGA-39-5029-01	0.778	1170.59
TCGA-50-5044-01	0	1710.57	TCGA-39-5028-01	0	779.008
TCGA-4B-A93V-01	0	1158.4	TCGA-39-5027-01	0	457.229
TCGA-49-AARR-01	1.5308	2569.55	TCGA-39-5024-01	0.2845	1150.91
TCGA-49-AARQ-01	15.1685	14908.4	TCGA-39-5022-01	32.1585	1889.88
TCGA-49-AARO-01	0	2634.59	TCGA-39-5021-01	0.5168	646.387
TCGA-49-AARN-01	8.209	2260.77	TCGA-39-5019-01	0.2493	760.663
TCGA-49-AARE-01	0	974.201	TCGA-39-5016-01	0.3571	873.064
TCGA-49-AAR9-01	0	1110.89	TCGA-39-5011-01	0	1312.36
TCGA-49-AAR4-01	0	3244.58	TCGA-37-A5EN-01	0	928.093
TCGA-49-AAR3-01	0.8711	2425.9	TCGA-37-A5EL-01	0	893.321
TCGA-49-AAR2-01	141.063	3901.38	TCGA-37-5819-01	1.1342	531.354
TCGA-49-AAR0-01	2.9747	2659.74	TCGA-37-4141-01	4.4374	682.755
TCGA-49-AAQV-01	0	1785.84	TCGA-37-4135-01	0	1071.51
TCGA-49-6767-01	0	1156.17	TCGA-37-4133-01	0.4375	436.911
TCGA-49-6761-01	0	919.025	TCGA-37-4132-01	1.7833	1178.2
TCGA-49-6745-01	0	722.609	TCGA-37-4130-01	302.329	1692.65
TCGA-49-6744-01	1.5275	1177.73	TCGA-37-4129-01	0	1058.56
TCGA-49-6743-01	0.5426	645.323	TCGA-37-3792-01	0	992.377
TCGA-49-6742-01	26.8456	930.574	TCGA-37-3789-01	0	1075.61
TCGA-49-4514-01	421.574	1104.3	TCGA-37-3783-01	0	1243.15
TCGA-49-4512-01	0	1625.56	TCGA-34-A5IX-01	0	904.257
TCGA-49-4510-01	0	1508.63	TCGA-34-8456-01	0	659.623
TCGA-49-4507-01	0.3459	1518.61	TCGA-34-8455-01	0	1336.49
TCGA-49-4506-01	195.403	1884.45	TCGA-34-8454-01	2.0644	1638.72
TCGA-49-4505-01	0	2269.5	TCGA-34-7107-01	0	485.414
TCGA-49-4501-01	8.4544	1727.35	TCGA-34-5929-01	8.0261	619.327
TCGA-49-4494-01	0.5679	951.322	TCGA-34-5928-01	0.9123	970.028
TCGA-49-4490-01	10.2967	1465.62	TCGA-34-5927-01	0	426.892
TCGA-49-4488-01	0.7338	1049.8	TCGA-34-5241-01	0	801.568
TCGA-49-4487-01	22.7464	1016.22	TCGA-34-5240-01	0	328.013
TCGA-49-4486-01	1.6625	1139.05	TCGA-34-5239-01	0	937.705
TCGA-44-A4SU-01	0	3142.35	TCGA-34-5236-01	0	444.715
TCGA-44-A4SS-01	0	2157.73	TCGA-34-5234-01	2.454	1387.26
TCGA-44-A47G-01	0	2321.16	TCGA-34-5232-01	0.5422	1612.3
TCGA-44-A47B-01	0	1585.41	TCGA-34-5231-01	0.7231	532.498
TCGA-44-A47A-01	0	2101.18	TCGA-34-2608-01	0.3599	980.401
TCGA-44-A479-01	5.6754	1304.14	TCGA-34-2600-01	0	736.919
TCGA-44-8120-01	0.841	555.068	TCGA-34-2596-01	0.363	426.264
TCGA-44-8119-01	0	586.306	TCGA-33-AASL-01	0.6197	947.718
TCGA-44-8117-01	0	913.034	TCGA-33-AASJ-01	0	501.348

TCGA-44-7672-01	0	1284.56	TCGA-33-AASI-01	0	2011.82
TCGA-44-7671-01	0	521.027	TCGA-33-AASD-01	0.4484	1153.08
TCGA-44-7670-01	5.2554	1582.04	TCGA-33-AASB-01	0	651.487
TCGA-44-7669-01	0.3067	681.157	TCGA-33-AAS8-01	0	732.362
TCGA-44-7667-01	0	275.573	TCGA-33-A5GW-01	0	530.263
TCGA-44-7662-01	0	1023.91	TCGA-33-A4WN-01	1.6	489.54
TCGA-44-7661-01	0.3123	940.334	TCGA-33-6738-01	1.5958	1192.89
TCGA-44-7660-01	0	914.209	TCGA-33-4589-01	1.2041	683.305
TCGA-44-7659-01	0.7474	1012.44	TCGA-33-4587-01	0.2675	370.463
TCGA-44-6779-01	0	1152.56	TCGA-33-4586-01	0	226.482
TCGA-44-6778-01	4.4416	1453.87	TCGA-33-4583-01	0	454.559
TCGA-44-6777-01	1.0521	1109.47	TCGA-33-4582-01	0	1583.78
TCGA-44-6776-01	0	794.26	TCGA-33-4566-01	0	341.053
TCGA-44-6775-01	8.5815	1125.37	TCGA-33-4547-01	0	868.708
TCGA-44-6774-01	0.9294	634.312	TCGA-33-4538-01	0	411.597
TCGA-44-6148-01	3.7498	1101.4	TCGA-33-4533-01	0	259.491
TCGA-44-6146-01	0.4513	1583.64	TCGA-33-4532-01	1.0846	313.955
TCGA-44-6145-01	233.733	1712.91	TCGA-22-A5C4-01	0.6836	4483.4
TCGA-44-5645-01	0.5737	1427.62	TCGA-22-5492-01	0.261	1007.11
TCGA-44-5644-01	1.995	726.264	TCGA-22-5491-01	0	242.218
TCGA-44-5643-01	0	676.952	TCGA-22-5489-01	0	1081.71
TCGA-44-4112-01	0	741.478	TCGA-22-5485-01	0	583.651
TCGA-44-3919-01	0.6504	1412.19	TCGA-22-5483-01	0.2371	462.422
TCGA-44-3918-01	0	1631.49	TCGA-22-5482-01	0.3122	485.716
TCGA-44-3398-01	0.4283	1251.22	TCGA-22-5481-01	0	1147.29
TCGA-44-3396-01	0	1582.26	TCGA-22-5480-01	0	2569.62
TCGA-44-2668-01	0	1194.39	TCGA-22-5479-01	0.4439	586.545
TCGA-44-2666-01	43.374	1289.13	TCGA-22-5478-01	1.0529	769.066
TCGA-44-2665-01	0.9488	1159.15	TCGA-22-5477-01	0	402.935
TCGA-44-2662-01	12.5685	1542.88	TCGA-22-5474-01	0.2734	778.256
TCGA-44-2659-01	0.9807	1164.57	TCGA-22-5473-01	0	687.386
TCGA-44-2657-01	1.3423	2869.5	TCGA-22-5472-01	0.6952	916.879
TCGA-44-2656-01	1.0839	1810.96	TCGA-22-5471-01	0	1368.19
TCGA-44-2655-01	1.0359	817.926	TCGA-22-4613-01	0	503.204
TCGA-38-A44F-01	3.9954	3946.73	TCGA-22-4609-01	0	1039.27
TCGA-38-7271-01	1.5186	1641.97	TCGA-22-4607-01	0.7454	724.849
TCGA-38-6178-01	0	866.958	TCGA-22-4605-01	1.119	577.678
TCGA-38-4632-01	0	1322.05	TCGA-22-4604-01	0	1004.19
TCGA-38-4631-01	0	432.975	TCGA-22-4601-01	0	931.767
TCGA-38-4628-01	3.4766	1036.98	TCGA-22-4599-01	0	966.129
TCGA-38-4627-01	0.7297	859.413	TCGA-22-4596-01	1.7233	1568.79
TCGA-38-4626-01	1.5836	1675.13	TCGA-22-4595-01	0.3355	665.277
TCGA-38-4625-01	0.5632	591.999	TCGA-22-4594-01	0	924.744
TCGA-35-5375-01	1.1099	1094.11	TCGA-22-4593-01	0	183.71

TCGA-35-4123-01	0	2362.27	TCGA-22-4591-01	0	417.137
TCGA-35-4122-01	66.8342	1242.27	TCGA-22-1017-01	0.7711	956.465
TCGA-35-3615-01	3.2489	827.531	TCGA-22-1016-01	0	1138.43
TCGA-05-5715-01	27.3277	1351.09	TCGA-22-1012-01	0	424.93
TCGA-05-5429-01	566.899	2506.45	TCGA-22-1011-01	0.551	1157.47
TCGA-05-5428-01	0	764.683	TCGA-22-1005-01	118.83	2989.82
TCGA-05-5425-01	579.186	1282.84	TCGA-22-1002-01	1.4091	911.479
TCGA-05-5423-01	0.7326	1110.78	TCGA-22-1000-01	0	1576.47
TCGA-05-4434-01	15.1481	1562.86	TCGA-21-5787-01	0	1035.03
TCGA-05-4433-01	0.8264	1681.4	TCGA-21-5786-01	0	335.853
TCGA-05-4432-01	4.3265	854.973	TCGA-21-5784-01	0	848.165
TCGA-05-4430-01	0.638	925.735	TCGA-21-5783-01	26.1279	514.135
TCGA-05-4427-01	0.5045	795.459	TCGA-21-5782-01	3.3708	908.416
TCGA-05-4426-01	0	1636.25	TCGA-21-1082-01	0.4059	1137.81
TCGA-05-4425-01	0	1244.47	TCGA-21-1081-01	0	689.425
TCGA-05-4424-01	1.4299	499.485	TCGA-21-1080-01	0.4019	304.441
TCGA-05-4422-01	356.469	2303.85	TCGA-21-1079-01	0	402.257
TCGA-05-4420-01	0.7889	640.518	TCGA-21-1076-01	0	1857.59
TCGA-05-4418-01	256.585	1290.98	TCGA-21-1075-01	0.7117	107.712
TCGA-05-4417-01	0.6354	874.109	TCGA-21-1072-01	0	137.817
TCGA-05-4415-01	0	224.707	TCGA-21-1071-01	0	537.865
TCGA-05-4410-01	0.7645	1041.31	TCGA-18-5592-01	0	266.43
TCGA-05-4405-01	0	922.462	TCGA-18-4721-01	0	480.799
TCGA-05-4403-01	1.6681	1619.84	TCGA-18-4086-01	0	586.652
TCGA-05-4402-01	1.9302	1215.66	TCGA-18-4083-01	0	827.956
TCGA-05-4398-01	0	1431.79	TCGA-18-3421-01	1.1338	660.249
TCGA-05-4397-01	39.757	1870.5	TCGA-18-3419-01	0	1318.58
TCGA-05-4396-01	448.825	636.679	TCGA-18-3417-01	0	499.252
TCGA-05-4395-01	0.2684	752.313	TCGA-18-3416-01	0	3290.18
TCGA-05-4390-01	0	784.595	TCGA-18-3415-01	0	634.543
TCGA-05-4389-01	0	1268.11	TCGA-18-3414-01	0	943.275
TCGA-05-4384-01	1.2217	1228.12	TCGA-18-3412-01	0.3912	649.847
TCGA-05-4382-01	0.4786	1302.17	TCGA-18-3411-01	0	573.533
TCGA-05-4250-01	0	832.44	TCGA-18-3410-01	0	1574.99
TCGA-05-4249-01	0.322	741.672	TCGA-18-3408-01	0.5932	79.8992
TCGA-05-4244-01	0	985.36	TCGA-18-3407-01	0	582.126
TCGA-S2-AA1A-01	14.4143	3775.57	TCGA-18-3406-01	0	854.677
TCGA-O1-A52J-01	0	1141.36	TCGA-22-0944-01	0	288.752
TCGA-NJ-A7XG-01	0.4579	1571.99	TCGA-22-0940-01	0	1064.28
TCGA-NJ-A55R-01	245.81	1256.94	TCGA-XC-AA0X-01	0	1418.13
TCGA-NJ-A55O-01	28.0047	2295.65	TCGA-O2-A5IB-01	0.2662	351.68
TCGA-NJ-A55A-01	0	1641.6	TCGA-O2-A52W-01	0	939.049
TCGA-NJ-A4YQ-01	0	2352.66	TCGA-O2-A52V-01	0	775.926
TCGA-NJ-A4YP-01	73.1707	1769.21	TCGA-O2-A52S-01	0	618.361

TCGA-NJ-A4YI-01	2.6855	1677.89	TCGA-O2-A52Q-01	0	2484.05
TCGA-NJ-A4YG-01	1.7321	1674.97	TCGA-O2-A52N-01	0	1895.84
TCGA-NJ-A4YF-01	0	468.791	TCGA-NK-A7XE-01	0	1447.46
TCGA-MP-A5C7-01	0	1248.83	TCGA-NK-A5CX-01	0.4891	1382.7
TCGA-MP-A4TK-01	0.4953	1917.73	TCGA-NK-A5CT-01	0	1440.75
TCGA-MP-A4TJ-01	0.8734	2178.21	TCGA-NK-A5CR-01	0	380.733
TCGA-MP-A4TI-01	0	3380.58	TCGA-NC-A5HT-01	0.3536	773.957
TCGA-MP-A4TH-01	0.9225	2080.9	TCGA-NC-A5HR-01	0.5814	910.843
TCGA-MP-A4TF-01	0.8157	1947.81	TCGA-NC-A5HQ-01	0.4579	1838.7
TCGA-MP-A4TE-01	0	578.242	TCGA-NC-A5HP-01	0	755.535
TCGA-MP-A4TD-01	10.3499	1456.48	TCGA-NC-A5HO-01	0	856.551
TCGA-MP-A4TC-01	0	1884.19	TCGA-NC-A5HN-01	0	228.837
TCGA-MP-A4TA-01	0.8079	1732.86	TCGA-NC-A5HM-01	0.5426	1019.77
TCGA-MP-A4T9-01	0.3982	1643.01	TCGA-NC-A5HL-01	0	1464.75
TCGA-MP-A4T8-01	0.8399	950.492	TCGA-NC-A5HK-01	0	1881.21
TCGA-MP-A4T7-01	163.212	1717.68	TCGA-NC-A5HJ-01	8.8129	1969.08
TCGA-MP-A4T6-01	0	6288.79	TCGA-NC-A5HI-01	0	418.779
TCGA-MP-A4T4-01	1.9333	3403.17	TCGA-NC-A5HH-01	0	682.529
TCGA-MP-A4SY-01	2.1646	1248.35	TCGA-NC-A5HG-01	0	789.155
TCGA-MP-A4SW-01	0	3452.8	TCGA-NC-A5HF-01	125.062	1584.83
TCGA-MP-A4SV-01	0.4275	1455.83	TCGA-NC-A5HE-01	0	2925.58
TCGA-MN-A4N5-01	0	2229.57	TCGA-NC-A5HD-01	0.5701	1250.44
TCGA-MN-A4N4-01	0	1303.81	TCGA-MF-A522-01	0.4266	169.736
TCGA-MN-A4N1-01	0	2895.06	TCGA-LA-A7SW-01	2.1805	526.677
TCGA-L9-A8F4-01	0	2303.17	TCGA-LA-A446-01	0.4183	590.296
TCGA-L9-A7SV-01	0	1371.09	TCGA-L3-A524-01	0.9421	399.152
TCGA-L9-A743-01	0	2202.29	TCGA-L3-A4E7-01	1.4171	3346.22
TCGA-L9-A5IP-01	0	668.655	TCGA-J1-A4AH-01	0	661.199
TCGA-L9-A50W-01	0	2448.56	TCGA-98-A53J-01	0	557.242
TCGA-L9-A444-01	0.7593	2802.6	TCGA-98-A53I-01	0.8522	1101.45
TCGA-L9-A443-01	0.5616	1237.64	TCGA-98-A53H-01	1.2381	1848.27
TCGA-L4-A4E6-01	4.4495	4321.08	TCGA-98-A53D-01	0.4819	2263.96
TCGA-L4-A4E5-01	0	1403.48	TCGA-98-A53C-01	9.5557	2398.8
TCGA-J2-A4AG-01	1.1257	1776.69	TCGA-98-A53B-01	0	1405.33
TCGA-J2-A4AE-01	1.182	2207.96	TCGA-98-A53A-01	0	1449.67
TCGA-J2-A4AD-01	0.812	755.794	TCGA-98-A539-01	0	810.746
TCGA-J2-8194-01	0	565.382	TCGA-98-A538-01	0	683.145
TCGA-J2-8192-01	0	1181.63	TCGA-98-8023-01	0	2567.01
TCGA-99-AA5R-01	1.0858	3188.6	TCGA-98-8022-01	20.203	2044.92
TCGA-99-8033-01	0.7812	1541.11	TCGA-98-8021-01	0.564	774.766
TCGA-99-8032-01	0	885.773	TCGA-98-8020-01	0	414.365
TCGA-99-8028-01	0.4307	2967.43	TCGA-98-7454-01	1.4263	750.325
TCGA-99-8025-01	0.3332	928.271	TCGA-96-A4JL-01	0.8358	1615.19
TCGA-99-7458-01	1.9863	1170.5	TCGA-96-8170-01	0.4161	1629.15

TCGA-97-A4M7-01	3.0082	2632.87	TCGA-96-8169-01	0.3818	2291.08
TCGA-97-A4M6-01	0	3146.91	TCGA-96-7545-01	0.3445	403.548
TCGA-97-A4M5-01	57.3702	2129.35	TCGA-96-7544-01	0	436.58
TCGA-97-A4M3-01	0.5058	1277.56	TCGA-94-A5I6-01	0	525.153
TCGA-97-A4M2-01	2.4082	4391.99	TCGA-94-A5I4-01	0.5728	738.671
TCGA-97-A4M1-01	1.9579	3575.76	TCGA-94-A4VJ-01	0	2018.86
TCGA-97-A4M0-01	0	3155	TCGA-94-8491-01	0	1581.35
TCGA-97-A4LX-01	0	2513.48	TCGA-94-8490-01	0.4163	522.331
TCGA-97-8552-01	1.2195	3295.32	TCGA-94-8035-01	0	473.878
TCGA-97-8547-01	56.6861	1006.43	TCGA-94-7943-01	0	470.276
TCGA-97-8179-01	1.1641	829.643	TCGA-94-7557-01	0	465.81
TCGA-97-8177-01	1.717	2198.04	TCGA-94-7033-01	0.4257	1300.54
TCGA-97-8176-01	127.843	803.544	TCGA-92-8065-01	0	1548.71
TCGA-97-8175-01	74.7826	3098.69	TCGA-92-8064-01	0	1576.83
TCGA-97-8174-01	2.4639	1476.94	TCGA-92-8063-01	0.6219	748.532
