# Supplementary figures and images for: FXR shapes an immunosuppressive microenvironment in PD-L1lo/– non-small cell lung cancer by upregulating HVEM
Source: JCI Insight. 2025 Sep 23;10(18):e190716. doi: 10.1172/jci.insight.190716 (PMC12487857; doi:10.1172/jci.insight.190716)

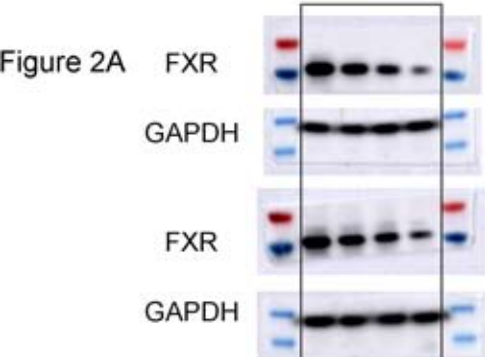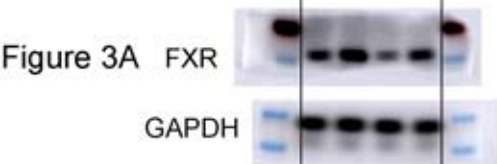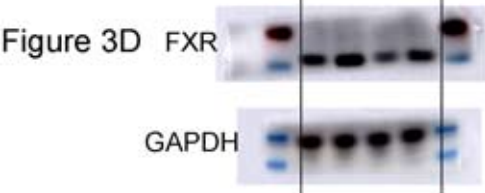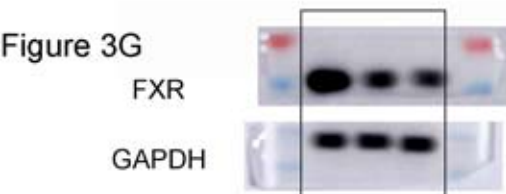

Supplementary

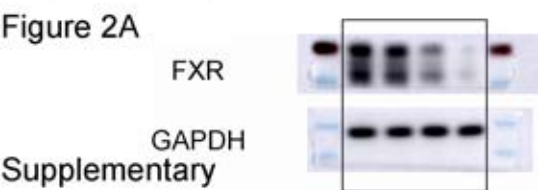

Supplementary

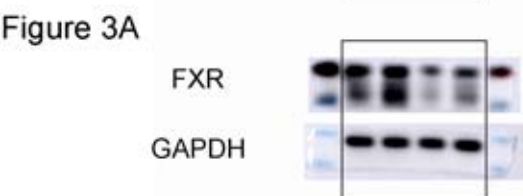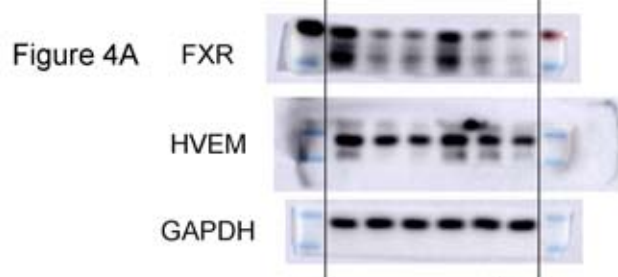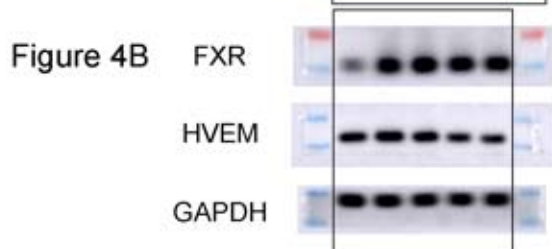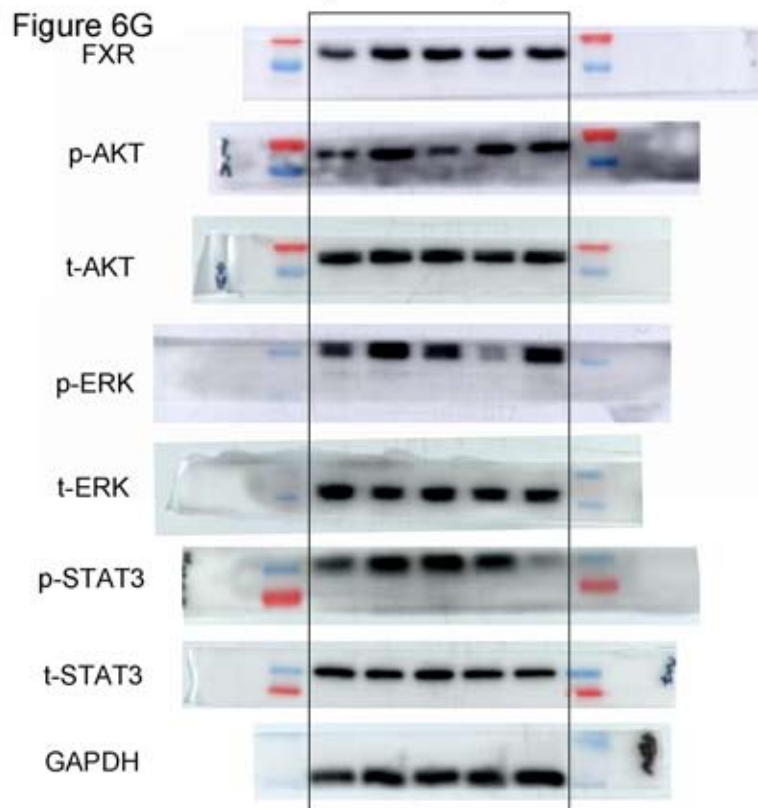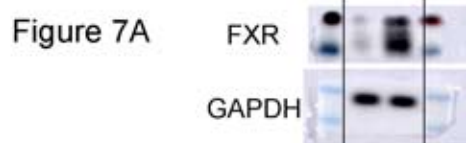

Supplement: Unedited blot and gel images [file jciinsight-10-190716-s201.pdf]
